# Cardiomyocyte Cell-Cycle Regulation in Neonatal Large Mammals: Single Nucleus RNA-Sequencing Data Analysis *via* an Artificial-Intelligence–Based Pipeline

**DOI:** 10.3389/fbioe.2022.914450

**Published:** 2022-07-04

**Authors:** Thanh Nguyen, Yuhua Wei, Yuji Nakada, Yang Zhou, Jianyi Zhang

**Affiliations:** ^1^ Department of Biomedical Engineering, University of Alabama at Birmingham, Birmingham, AL, United States; ^2^ Cardiovascular Diseases, Department of Medicine, University of Alabama at Birmingham, Birmingham, AL, United States

**Keywords:** single-nucleus RNA-sequencing, heart, infarct, cardiomyocytes, cell cycle, autoencoder

## Abstract

Adult mammalian cardiomyocytes have very limited capacity to proliferate and repair the myocardial infarction. However, when apical resection (AR) was performed in pig hearts on postnatal day (P) 1 (AR_P1_) and acute myocardial infarction (MI) was induced on P28 (MI_P28_), the animals recovered with no evidence of myocardial scarring or decline in contractile performance. Furthermore, the repair process appeared to be driven by cardiomyocyte proliferation, but the regulatory molecules that govern the AR_P1_-induced enhancement of myocardial recovery remain unclear. Single-nucleus RNA sequencing (snRNA-seq) data collected from fetal pig hearts and the hearts of pigs that underwent AR_P1_, MI_P28_, both AR_P1_ and MI, or neither myocardial injury were evaluated *via* autoencoder, cluster analysis, sparse learning, and semisupervised learning. Ten clusters of cardiomyocytes (CM1–CM10) were identified across all experimental groups and time points. CM1 was only observed in AR_P1_ hearts on P28 and was enriched for the expression of T-box transcription factors 5 and 20 (TBX5 and TBX20, respectively), Erb-B2 receptor tyrosine kinase 4 (ERBB4), and G Protein-Coupled Receptor Kinase 5 (GRK5), as well as genes associated with the proliferation and growth of cardiac muscle. CM1 cardiomyocytes also highly expressed genes for glycolysis while lowly expressed genes for adrenergic signaling, which suggested that CM1 were immature cardiomyocytes. Thus, we have identified a cluster of cardiomyocytes, CM1, in neonatal pig hearts that appeared to be generated in response to AR injury on P1 and may have been primed for activation of CM cell-cycle activation and proliferation by the upregulation of TBX5, TBX20, ERBB4, and GRK5.

## Introduction

Mammalian cardiomyocytes exit the cell cycle during the perinatal period and lose the ability to proliferate; thus, the hearts of mammals are unable to repair the damage caused by myocardial injury that occurs more than 2 days after birth (Porrello et al., 2011; Lam and Sadek, 2018; Ye et al., 2018; Zhu et al., 2018). However, when apical resection (AR) was performed in pig hearts on postnatal day (P) 1 (AR_P1_), and acute myocardial infarction (MI) was induced on P28 (MI_P28_), the animals completely recovered with no evidence of myocardial scarring or decline in contractile performance by P56, whereas the hearts of animals that underwent MI on P28 without previous AR injury displayed significant fibrosis and declines in contractile activity (Zhao et al., 2020). Furthermore, the repair process appeared to be driven by the proliferation of cardiomyocytes, and comparative analyses of bulk and single-nuclei RNA sequencing (snRNA-seq) data from the hearts of animals that underwent AR_P1_ only, MI_P28_ only, or both AR_P1_ and MI_P28_ (AR_P1_MI_P28_), as well as uninjured (CTL) and fetal pig hearts, suggested that signaling pathways associated with cell-cycle activity, glycolytic metabolism, and declines in DNA damage were upregulated in the cardiomyocytes of AR_P1_MI_P28_ hearts (Zhang et al., 2020; Zhao et al., 2020; Nakada et al., 2022). Also, in our previous study using snRNA-seq data (Nakada et al., 2022), we found a novel cardiomyocyte subpopulation marked by coupregulation of Nebulin (NEB) and Pyruvate Kinase M1/2 (PKM), which uniquely appeared in regenerative AR_P1_MI_P28_ heart on postnatal day P35. On the other hand, how AR_P1_ cardiomyocytes differed from CTL ones such that they responded differently following MI_P28_ injury was not thoroughly examined. For the studies presented in this report, we collected more snRNA-seq data from the cardiac tissues of additional animals and then analyzed our expanded dataset with an artificial-intelligence–based pipeline for deeper understanding on which regulatory molecules and signaling pathways contributed to the AR_P1_-associated enhancement of myocardial regeneration, and which cardiomyocyte subsets highly utilizes these regulators. We expanded the snRNA-seq dataset by obtaining a new MI_P28_-only group on postnatal days P30, P35, and P42 and more AR_P1_-MI_P28_ pigs at P30, P35, and P42. In addition, an artificial-intelligence technique was developed and applied for high-dimensional snRNA-seq data using a much smaller number of dimensions (Wang et al., 2016) and consequently improved snRNA-seq data clustering findings.

## Results

### Autoencoding and Cluster Analysis Identified 10 Cardiomyocyte Populations in the Hearts of Fetal, AR_P1_, MI_P28_, and AR_P1_MI_P28_ Animals

Our complete dataset encompassed the results from snRNA-seq analyses of myocardial tissues in animals that underwent AR_P1_ only and were sacrificed on P28 and P56 (AR_P1_-P28 and ARP1-P56, respectively); animals that underwent MI_P28_ only and were sacrificed on P30, P35, P42, and P56 (MI_P28_-P30, -P35, -P42, and -P56, respectively); animals that underwent both AR_P1_ and MI_P28_ and were sacrificed on P30, P35, P42, and P56 (AR_P1_MI_P28_-P30, -P35, -P42, and -P56, respectively); CTL animals that were sacrificed on P1, P28, and P56 (CTL-P1, -P28, and -P56, respectively); and embryos obtained on embryonic day 80 (Fetal) (Supplementary Table S1). Tissues were collected from the border zone of infarction or the corresponding region of non-infarcted hearts, and nuclei with <500 or >25,000 unique molecular identifiers (UMIs), or with >25% mitochondrial UMIs were excluded from subsequent analyses. A total of 283,421 nuclei from 41 hearts were included in our analyses; 1,786 (median) genes and 31,736 (median) UMIs were captured per nucleus (Supplementary Table S2), and 129,991 of the nuclei were from cardiomyocytes. Data were aligned and normalized with the Seurat v.4 toolkit (Hao et al., 2021), and then embedded with an autoencoder before clustering and visualization *via* Uniform Manifold Approximation and Projection (UMAP) (McInnes et al., 2018; Meehan, 2021); this deep-learning–based method has outperformed other state-of-the-art approaches for unsupervised cluster analysis (Yang et al., 2019).

**FIGURE 1:**
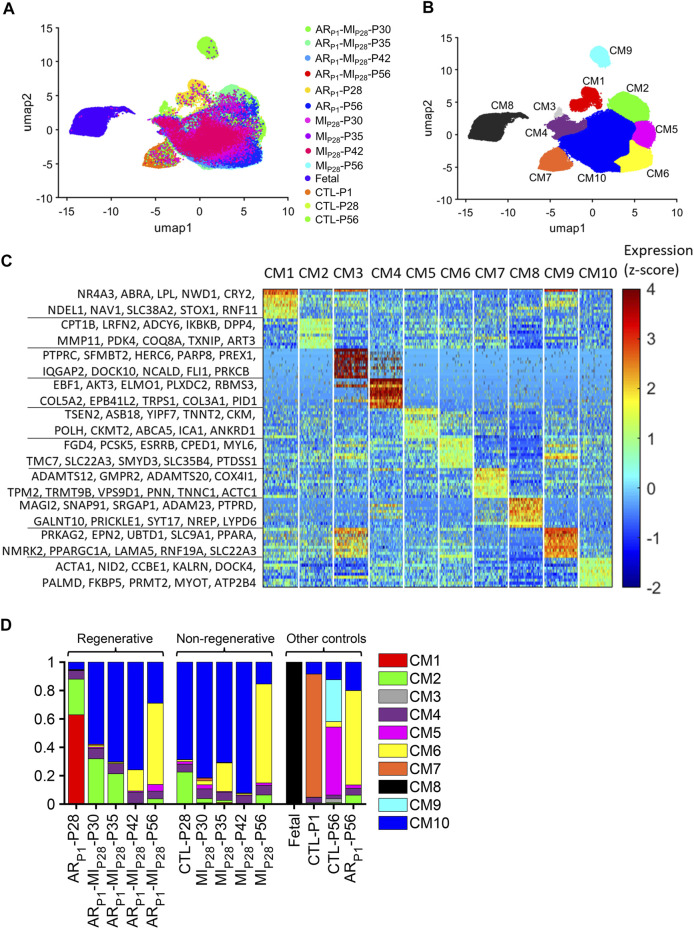
Cluster analysis identified 10 populations of cardiomyocytes in Fetal, CTL, AR_P1_, MI_P28_, and AR_P1_MI_P28_ hearts. **(A,B)** Cardiomyocyte snRNA-seq data were reduced to 10 dimensions with an autoencoder, processed *via* cluster analysis, visualized *via* UMAP, and displayed according to **(A)** experimental group and time point and **(B)** cluster (CM1–CM10). **(C)** The expression of genes that were explicitly associated with each cluster is displayed as a heat map. **(D)** The proportion of cardiomyocytes from each cluster is displayed for each experimental group and time point.

When data for all cell types were analyzed (Supplementary Figure S1), most of the clusters contained cells from multiple injury groups and time points, indicating that the results were not influenced by between-batch variation or sampling error, and each cluster was composed primarily of a single cell type (cardiomyocytes, smooth muscle cells, endothelial cells, fibroblasts, immune cells, or skeletal muscle–like cells. The skeletal muscle–like cluster uniquely expressed exclusive-skeletal-markers myosin light chain 3 (MYL3) (Hailstones and Gunning, 1990) and Myosin light chain kinase 2 (MYLK2) (Stull et al., 2011); meanwhile, it expresses cardiac Actin Alpha Cardiac Muscle 1 (ACTC1) and Myosin Heavy Chain 7 (MYH7). Cardiac muscle populations expressing both cardiomyocyte and skeletal muscle markers were reported in Clément et al. (1999). Also, the cardiac/skeletal muscle–like cluster upregulated both Nebulin (NEB) and Pyruvate Kinase M1/2 (PKM), which were consistent with the PKM+/NEB + cardiomyocyte subpopulation in our previous report (Nakada et al., 2022). Cardiomyocytes were distributed into 10 clusters (CM1–CM10) (Figures 1A, B), each of which was associated with a set of explicitly expressed marker genes (Figure 1C, Supplementary Tables S3–S4).

Cardiomyocytes in the CM1 cluster were found almost exclusively in AR_P1_-P28 hearts, where they comprised 62.91% of the total cardiomyocyte population (Figure 1D). The CM2 cluster included a substantial proportion of cardiomyocytes from CTL (22.53%) and AR_P1_ (25.11%) hearts at P28, as well as AR_P1_MI_P28_ hearts at P30 (31.77%) and P35 (21.17%). CM3 cardiomyocytes were present only in CTL-P56 hearts, where they comprised just 2.63% of all cardiomyocytes and expressed elevated levels of genes associated with cell differentiation. Small numbers of CM4 cardiomyocytes, which expressed high levels of collagen [Collagen Type V Alpha 2 Chain (COL5A2) and Collagen Type III Alpha 1 Chain (COL3A1)] and genes that regulate the pluripotency of stem cells, including APC (Kielman et al., 2002), PIK3CA (Jeong et al., 2017), MAPK1 (Lu et al., 2008), and JARID2 (Landeira et al., 2010) (Supplementary Figure S2A), were present in all hearts. Cardiomyocytes from CM5 were only in CTL-P56 hearts and explicitly expressed genes that drive cardiomyocyte maturation, such as Ankyrin Repeat And SOCS Box Containing 18 (ASB18), Yip1 Domain Family Member 7 (YIPF7), Creatine Kinase, M-Type (CKM), and Creatine Kinase, Mitochondrial 2 (CKMT2) (Uosaki et al., 2015; Guo and Pu, 2020). CM6 included the majority (57.16%–69.69%) of cardiomyocytes from all injury groups (AR_P1_, MI_P28_, and AR_P1_MI_P28_) on P56 and was enriched for the expression of Z-disc and insulin-signaling genes. CM7 encompassed most (86.75%) of the cardiomyocytes in CTL-P1 hearts and was associated with elevated levels of genes that participate in the morphogenesis of cardiac muscle and myofibril assembly. The CM8 cluster included nearly all (99.69%) of the cardiomyocytes in fetal hearts, none of those from any other group or time point, and was enriched for genes that contribute to embryonic development. CM9 cardiomyocytes were present only in CTL-P56 hearts, where they comprised 29.51% of all cardiomyocytes, and expressed high levels of genes associated with the Z-disc, focal-adhesion, and other structural components of muscle cells. Finally, cardiomyocytes from the CM10 cluster were found in all nonfetal groups at all time points and included the majority (57.85%–92.16%) of cardiomyocytes in MI_P28_ and AR_P1_MI_P28_ hearts from P30-P42. The pairwise similarities among these clusters were presented in Supplementary Figure S3.

### AR_P1_ and MI_P28_, Both Alone and in Combination, Promoted Cardiomyocyte Cell-Cycle Activity for Approximately Two Weeks After Myocardial Infarction Induction

Sparse modeling (Bi et al., 2003; Huang et al., 2010; Chkifa et al., 2015; Zhang et al., 2014) enables researchers to extract relevant data from datasets that contain a large number of variables that do not contribute to the property being studied. When sparse modeling was used to evaluate the expression of cell-cycle genes in our cardiomyocyte snRNA-seq dataset (Supplementary Figure S4), our results are consistent with our previous report in (Nakada et al., 2022). Briefly, the proportion of high-cell-cycle in each phase of the cell cycle was the greatest in Fetal and CTL-P1 hearts. Also, these proportions in CTL-P1 hearts were higher than in hearts from any other postnatal group, which is consistent with the perinatal occurrence of cardiomyocyte cell-cycle arrest. However, cycling cardiomyocytes were much more common in AR_P1_ hearts at P28, and in both MI_P28_ and AR_P1_MI_P28_ hearts from P30-P42, than in CTL hearts at P28 or P56. Cell-cycle activity was also significantly more common in cardiomyocytes from AR_P1_MI_P28_ hearts than from MI_P28_ hearts on P35 (G1S: *p* = 1.06 × 10^−58^; S: *p* = 9.67 × 10^−12^; G2M: *p* = 7.44 × 10^−66^; M: *p* = 4.11 × 10^−7^) but not on P42, which suggests that AR on P1 promoted cardiomyocyte proliferation for approximately 1 week after MI induction on P28. Notably, this period of AR_P1_-enhanced proliferation coincided with a greater proportion of CM2 cardiomyocytes in AR_P1_MI_P28_ hearts.

### AR_P1_-P28 Hearts Contained a Cluster of Cardiomyocytes With a Latent Capacity for Myocardial Proliferation and Growth

**FIGURE 2:**
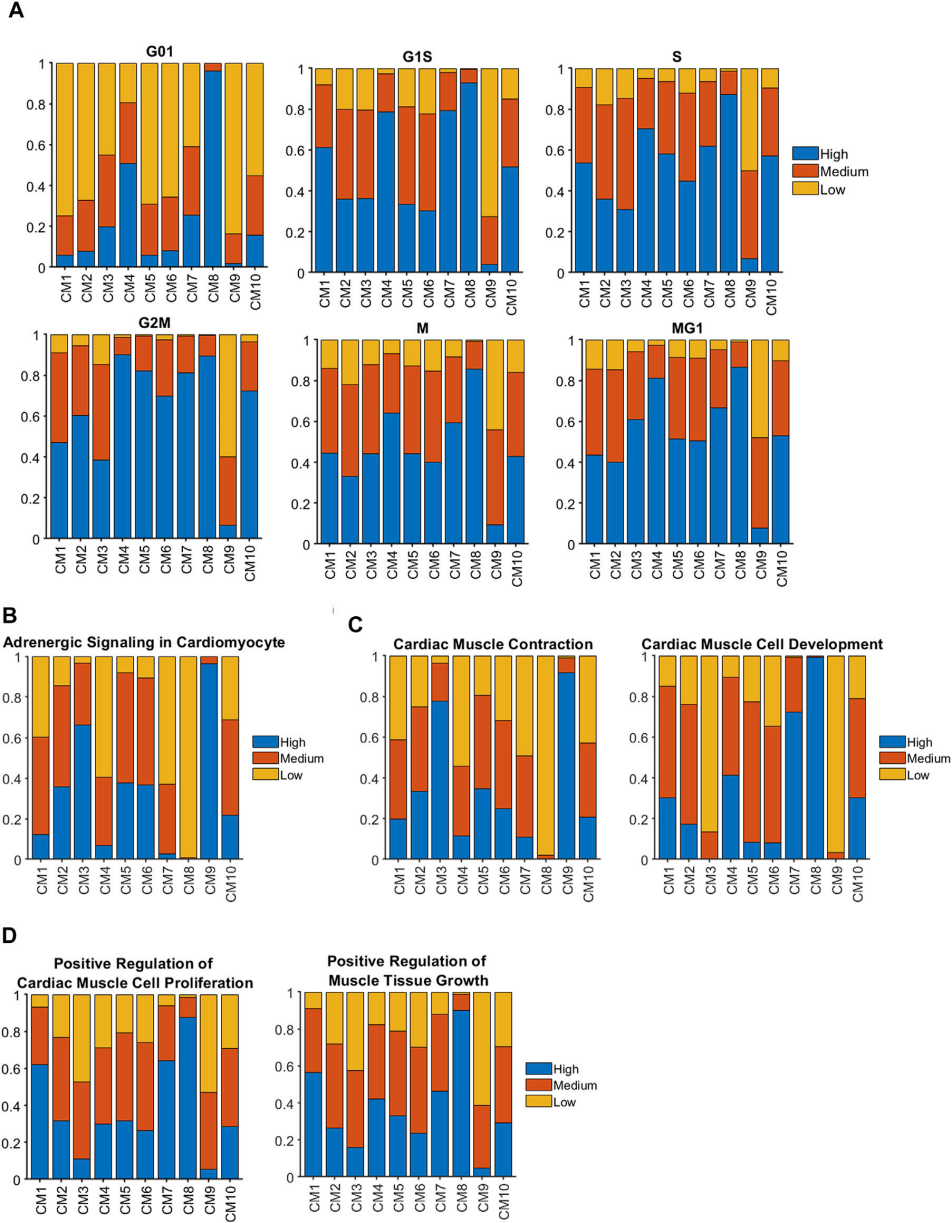
Genes that contribute to cell-cycle activity and muscle growth tended to be more highly expressed in clusters associated with regenerating hearts than in CTL hearts at P28 and afterward. Sparse analysis was conducted for the expression of genes associated with **(A)** the cell-cycle, **(B)** adrenergic signaling, **(C)** the contractile activity and development of cardiac muscle, and **(D)** cardiac-muscle cell proliferation and muscular growth; then, the proportion of cardiomyocytes with high, medium, or low levels of expression was calculated for each cluster.

When cell-cycle gene expression was evaluated for cardiomyocyte (CM1) clusters (Figure 2A), cycling activity tended to be highest in CM8 and lowest in CM9, which is consistent with our observation that these two clusters were almost exclusively associated with Fetal and CTL-P56 hearts, respectively. The proportion of cycling cells was also elevated among CM4 cardiomyocytes, which express high levels of pluripotency-maintenance genes (Mouse Genome Informatics, 2022) (Supplementary Figure S2B) and consequently, appear to have some progenitor-cell–like properties. However, CM1 cardiomyocytes, which were found only in AR_P1_ hearts at P28, were not especially more proliferative than CM9 (primarily in CTL-P56); for example, G2M (Mouse Genome Informatics, 2021a) [odds ratio (OR) = 2.66, *p* = 3.38 × 10^−76^] and MG1 (Cui et al., 2020) (OR = 1.45, *p* = 1.59 × 10^−21^) cells were less common in CM1 than in CM10, which comprised the bulk of cardiomyocytes in both MI_P28_ and AR_P1_MI_P28_ hearts during the 2 weeks after MI induction. Nevertheless, analyses of adrenergic signaling (KEGG: Adrenergic signaling in cardiomyocytes, 2021) (Figure 2B), cardiac-muscle contraction (Mouse Genome Informatics, 2021b), and cardiac-muscle–cell development (Gene Ontology: Cardiac Muscle Cell Development, 2021) (Figure 2C) indicated that CM1 cardiomyocytes were functionally immature, while genes associated with the proliferation (Mouse Genome Informatics, 2021c) (OR = 5.61, *p* < 10^−60^) and growth (Mouse Genome Informatics, 2021d) (OR = 4.35, *p* < 10^−60^) of cardiac muscle (Figure 2D) were more highly expressed in CM1 than in CM10. Collectively, these observations suggest that although CM1 cardiomyocytes themselves did not display exceptionally high levels of cell-cycle activity, they were still immature and might retain a latent capacity for proliferation that could have been reactivated by MI induction on P28.

### CM1 Cardiomyocytes Express Elevated Levels of T-Box Transcription Factors 5 and 20 (TBX5 and TBX20), Erb-B2 Receptor Tyrosine Kinase 4, and G Protein-Coupled Receptor Kinase 5

**FIGURE 3:**
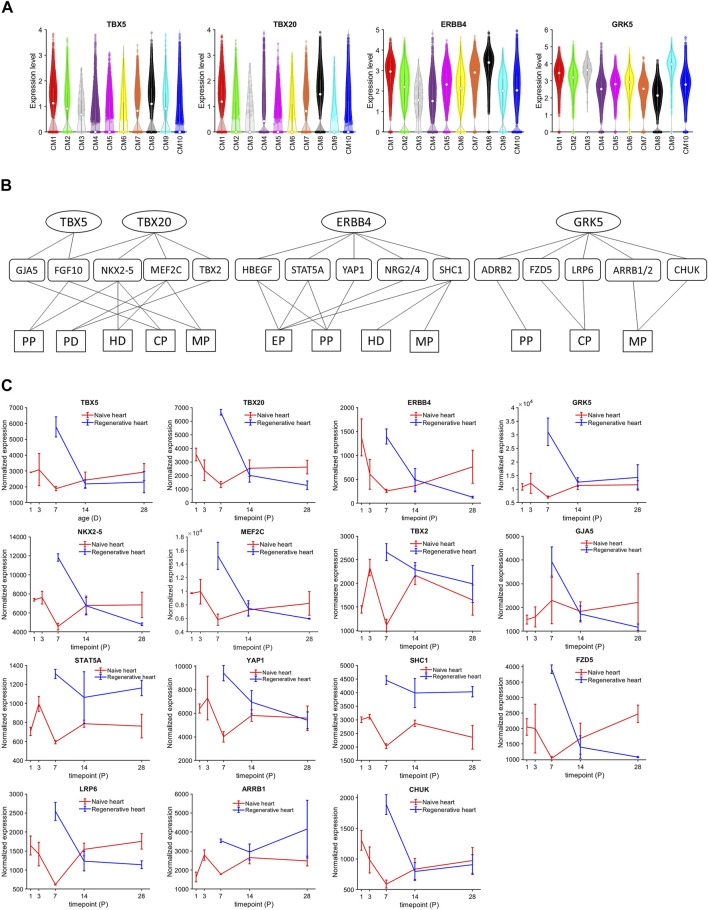
TBX5, TBX20, ERBB4, and GRK5 were consistently upregulated in CM1, CM7, and CM8 cardiomyocytes. **(A)** The abundance of TBX5, TBX20, ERBB4, and GRK5 RNA transcripts was measured in cardiomyocytes from each cluster and displayed in a violin plot. The expression was normalized according to the Seurat pipeline: the expression matrix was scaled by the ScaleData function with vars. to. regress set to nUMI and nGenes; then, the scaled expression was log-normalized. **(B)** The network of genes, cellular processes, and signaling pathways regulated by TBX5, TBX20, ERBB4, and GRK5 was evaluated with DAVID bioinformatics resources. Targeted genes were identified in the TRRUST v2 (TBX5 and TBX20) and STRING v11.5 (ERBB4 and GRK5) databases and functional/pathway data were obtained from the GOA, KEGG, and Reactome databases. PP, positive regulation of cell proliferation; PD, positive regulation of cardiac muscle cell differentiation; CP, canonical Wnt signaling pathway; MP, MAPK signaling pathway; Ep, ErbB signaling pathway. **(C)** The bulk RNAseq expressions of TBX5, TBX20, ERBB4, GRK5, NKX2-5, MEF2C, TBX2, GJA5, STAT5A, YAP1, SHC1, FZD5, LRP5, ARRB1, and CHUK in regenerative and naïve pig heart. The transcript counts were normalized using DeSeq2 pipeline.

CM1 cardiomyocytes were enriched for expression of the regulatory molecules TBX5 (*p* = 8.65 × 10^−184^), TBX20 (*p* = 2.97 × 10^−225^), and Erb-B2 receptor tyrosine kinase 4 (ERBB4) (*p* = 4.86 × 10^−186^) (Figure 3A). Notably, these three genes were also coupregulated in the clusters that were exclusively associated with Fetal hearts (CM8) and with CTL hearts on P1 (CM7), and previous reports have shown that disruptions of TBX5 (Misra et al., 2014; Maitra et al., 2009), TBX20 (Xiang et al., 2016; Chakraborty and Yutzey, 2012), or ERBB4 (Bersell et al., 2009) activity reduce cardiomyocyte proliferation in fetal and neonatal mouse hearts. Furthermore, the expression of G protein-coupled receptor kinase 5 (GRK5), which is associated with cardiac hypertrophy (Gold et al., 2012; Traynham et al., 2015), was upregulated in CM1 cardiomyocytes. Thus, we queried the TRRUSTv2 (Han et al., 2018) and STRING v11.5 (Szklarczyk et al., 2021) databases to identify the genes that are targeted by or interact with these four molecules, and then used the database for annotation, visualization, and integrated discovery (DAVID) bioinformatics resources (Huang et al., 2009) with the Gene Ontology Annotation (GOA) (Huntley et al., 2015), Kyoto Encyclopedia of Genes and Genomes (KEGG) (Kanehisa et al., 2017), and Reactome (Jassal et al., 2020) databases to characterize the network (Figure 3B) of biological processes and signaling pathways that may have been upregulated in CM1 cardiomyocytes. Collectively, our results identified increases in processes of cell proliferation (Mouse Genomic Informatics, 2021a) and cardiac-muscle–cell differentiation (Mouse Genome Informatics, 2021c), as well as in ErbB (Zhu et al., 2010), MAPK (Liu and Zhong, 2017), and canonical Wnt (Kwon et al., 2007) signaling (Supplementary Tables S5, S6). Intermediate molecules in the network included: 1) Fibroblast Growth Factor 10 (FGF10), which is activated by both TBX5 and TBX20, phosphorylates Forkhead Box O3 (FOXO3), and downregulates the cell-cycle inhibitor p27 (Rochais et al., 2014); 2) Gap Junction Protein Alpha 5 (GJA5), which is activated by TBX5 and contributes to the differentiation of induced-pluripotent stem cells into cardiomyocytes, endothelial cells, and other cardiac cell types (Paik et al., 2018); 3) Myocyte Enhancer Factor 2C (MEF2C) and NK2 Homeobox 5 (NKX2-5), which are upregulated by TBX20 and cooperatively participate in the embryonic development of mouse hearts (Vincentz et al., 2008; Materna et al., 2019); 4) the Hippo pathway effector Yes-associated Protein 1 (YAP1), which is activated by ERBB4 and regulates the cell cycle by interacting with transcription factors of the Transcriptional Enhanced Associate Domain (TEAD) family (Zhao et al., 2008; Yuan et al., 2020); and 5) Beta-2 Adrenergic Receptor (ADRB2), as well as downstream components of the canonical Wnt signaling pathway (Chen et al., 2009), which may reactivate proliferation in mature cardiomyocytes (Fan et al., 2018). Notably, bulk-RNA seq data (Zhang et al., 2020) cross-checking also showed the overexpression of TBX5, TBX20, ERBB4, GRK5, NKX2-5, MEF2C, TBX2, GJA5, STAT5A, YAP1, SHC1, FZD5, LRP6, ARRB1, and CHUK among regenerative hearts (Figure 3C). Here, these regenerative-hearts expressions on postnatal day 7 (P7) were even higher than they are in naïve-hearts P1 and P3.

### CM1 and CM2 Cardiomyocytes Were Enriched for the Expression of Genes That Contribute to Both Glycolysis and β Fatty Acid Oxidation

**FIGURE 4:**
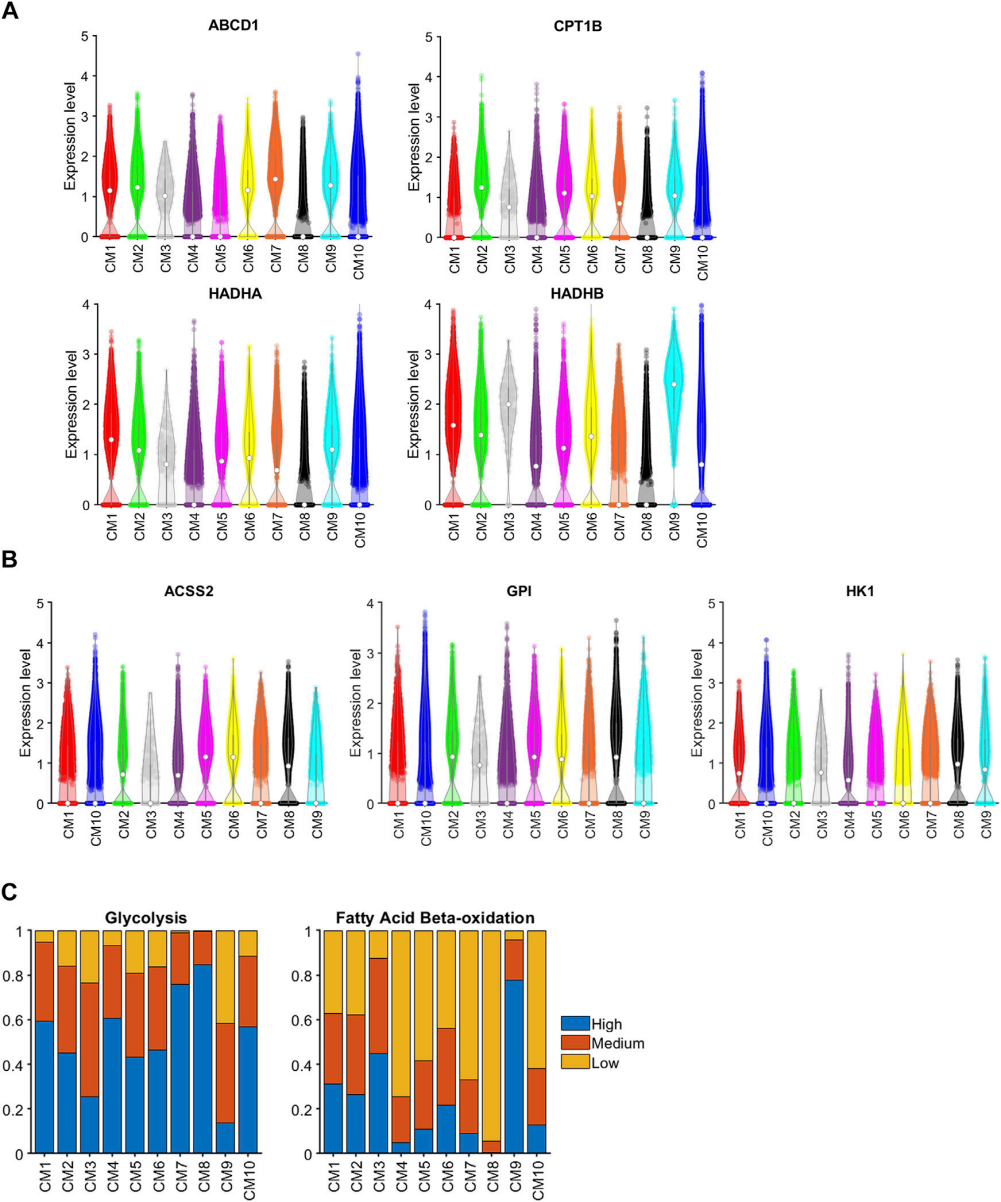
Genes associated with both glycolysis and βFAOX were upregulated in CM1 and CM2. The abundance of RNA transcripts for genes that contribute to **(A)** glycolysis (ABCD1, CPT1B, HADHA, and HADHB) and **(B)** βFAOX (ACSS2, GPI, and HK1) was measured in cardiomyocytes from each cluster and displayed in a violin plot. The expression was normalized according to the Seurat pipeline: the expression matrix was scaled by the ScaleData function with vars.to.regress set to nUMI and nGenes; then, the scaled expression was log-normalized. **(C)** Sparse analysis was conducted for the expression of glycolysis and βFAOX genes, and the proportion of cardiomyocytes with high, medium, or low levels of expression was calculated for each cluster.

As cardiomyocytes mature, the primary mechanism for ATP generation switches from glycolysis to beta fatty acid oxidation (β-FAOX) (Lopaschuk and Jaswal, 2010), which is consistent with our observation that glycolysis genes [Acyl-CoA Synthetase Short Chain Family Member 2 (ACSS2), Glucose-6-Phosphate Isomerase (GPI), and Hexokinase 1 (HK1) (Glycolysis, 2021)] were highly expressed in the CM8 cluster (i.e., fetal cardiomyocytes) but not in CM9 (i.e., the CTL-P56–exclusive cluster), while genes involved in β-FAOX [ATP Binding Cassette Subfamily D Member 1 (ABCD1) (van Roermund et al., 2011), Carnitine Palmitoyltransferase 1B (CPT1B) (Angelini et al., 2021), and Hydroxyacyl-CoA Dehydrogenase Trifunctional Multienzyme Complex Subunits Alpha (Miklas et al., 2019) and Beta (Sekine et al., 2021) (HADHA and HADHB, respectively)] were downregulated in CM8 and upregulated in CM9 (Figures 4A, B). Sparse-model analysis indicated that glycolysis (Glycolysis, 2021) was the dominant metabolic pathway in most other cardiomyocyte clusters. Compared to CM9 (primarily CTL-P56 cardiomyocytes), glycolysis markers were upregulated in CM1 (OR = 9.21, *p* < 10^−60^), CM2 (OR = 5.23, *p* < 10^−60^), and CM10 (OR = 8.28, p10^−60^), yet did not reach the CM7 (CTL-P1 exclusive) and CM8 (Fetal exclusive) level. In addition, compared to CM8, β-FAOX markers were upregulated in CM1 (OR = 128.77, *p* < 10^−60^), CM2 (OR = 102.75, *p* < 10^−60^), and in CM10 (OR = 41.68, *p* < 10^−60^), yet did not reach CM9 level, (Figure 4C). β-FAOX upregulation in CM10 was significantly less than in CM1 and CM2. Notably, the CM2 and CM10 clusters comprised most (∼90% or more) of the cardiomyocytes in AR_P1_MI_P28_ hearts during the first week after MI induction, and DAVID analysis indicated that the metabolism of CM2 but not CM10, cardiomyocytes was also supported by increases in insulin (KEGG: Insulin signaling pathway, 2021) and glucagon (KEGG: Insulin signaling pathway, 2021) signaling. Nevertheless, assessments of cell-cycle activity in the CM2 and CM10 clusters were similar (Figure 3A), so whether CM2 cardiomyocytes have a unique role in the AR_P1_-induced enhancement of myocardial repair and regeneration remains unclear.

### The Fate of CM1 Cardiomyocytes After Myocardial Infarction on P28 May Have Been Regulated by Protein Kinase AMP-Activated Noncatalytic Subunit Gamma 2, Nuclear Receptor Subfamily 4 Group a Member 3, and Activating Transcription Factor 3

**FIGURE 5:**
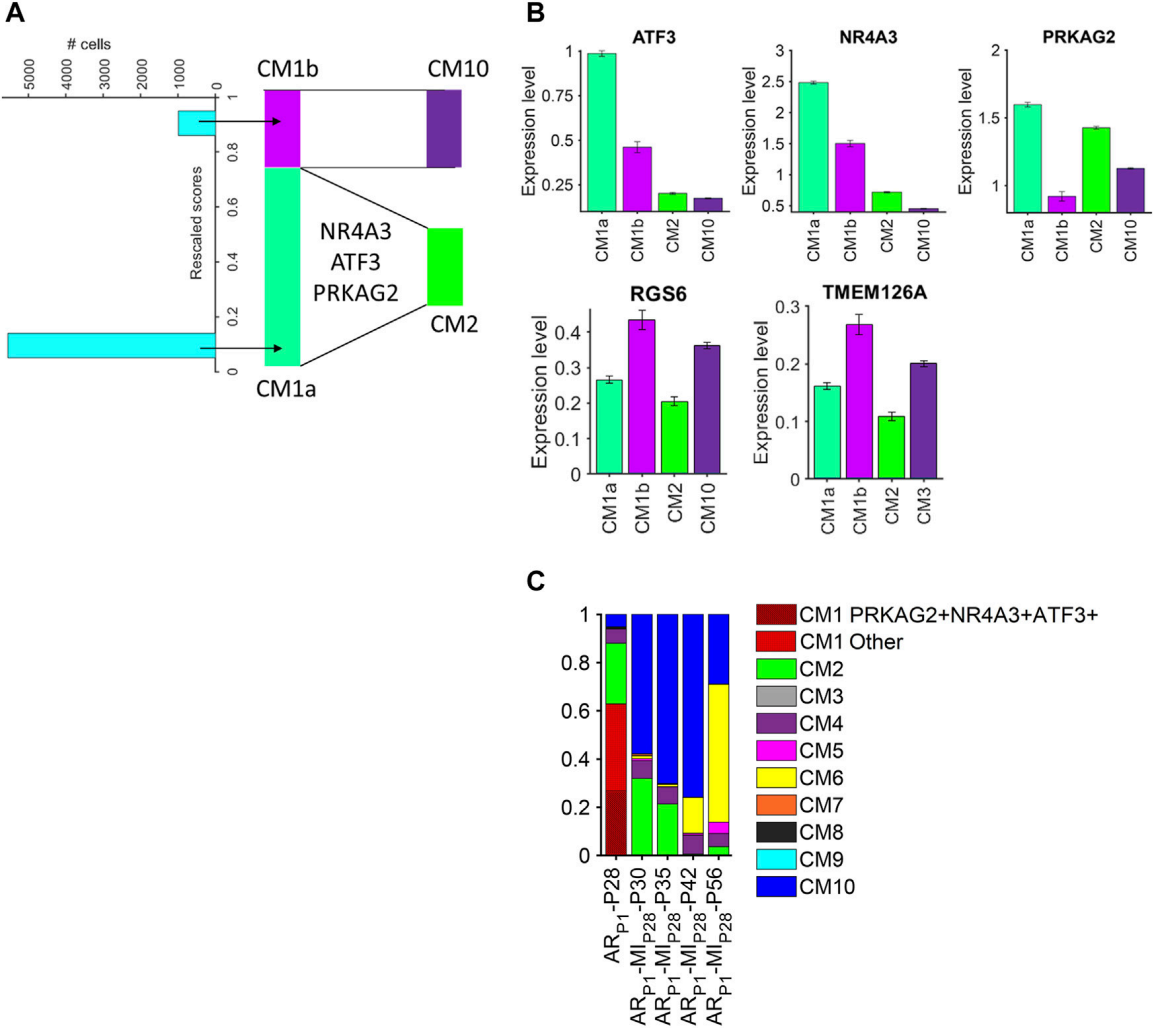
Cardiomyocytes in the CM1 cluster could be partitioned into CM2- and CM10-like subpopulations. **(A)** snRNA-seq data for CM1 cardiomyocytes in AR_P1_-P28 hearts and for CM2 and CM10 cardiomyocytes in AR_P1_MI_P28_-P30 hearts were evaluated with a semisupervised learning model to calculate a rescaled score for each CM1 cardiomyocyte. Cardiomyocytes with rescaled scores approaching 0 were designated CM1a (CM2-like) and cardiomyocytes with rescaled scores approaching 1 were designated CM1b (CM10-like). **(B)** The abundance of ATF3, NR4A3, PRKAG2, RGS6, and TMEM126A RNA transcripts was measured in CM1a, CM1b, CM2, and CM10 cardiomyocytes. **(C)** The proportion of PRKAG2+NR4A3+ATF3+ cardiomyocytes in CM1 (AR_P1_-P28), in comparison with CM2 proportions.

Because cardiomyocytes in the CM1 cluster appeared to retain some latent capacity for proliferation and were present almost exclusively in AR_P1_ hearts at P28, whereas the CM2 and CM10 clusters collectively encompassed most of the cardiomyocytes in AR_P1_MI_P28_ hearts at P30 and P35 and were more similar to CM1 than other AR_P1_MI_P28_ clusters (Supplementary Figure S3), we used a semisupervised machine-learning technique to investigate whether the induction of MI in hearts that had recovered from previous AR surgery could have triggered the transformation of CM1 cardiomyocytes into CM2 and CM10 cardiomyocytes. snRNA-seq data for each cardiomyocyte in the CM1 cluster were compared to data for CM2 and CM10 cardiomyocytes from AR_P1_MI_P28_ hearts on P30; then, the CM1 cardiomyocytes were distributed by semisupervised machine-learning into two subpopulations: CM1a or CM1b, which more closely resembled cardiomyocytes from the CM2 or CM10 clusters. (Figure 5A). Transcription factors that were differentially expressed between the CM1a and CM1b subpopulations (Figure 5B, Supplementary Table S7) included protein kinase AMP-activated noncatalytic subunit gamma 2 (PRKAG2), nuclear receptor subfamily 4 group a member 3 (NR4A3), and activating transcription factor 3 (ATF3), which were also more highly expressed in CM2 than in CM10 cardiomyocytes, and the expression of all three genes declined in ARP1MIP28 hearts after P35, which coincided with the disappearance of CM2 cardiomyocytes. Notably, PRKAG2 regulates both glycolysis and fatty acid oxidation (Hinson et al., 2016), which were uniquely coupregulated in CM1 and CM2 cardiomyocytes, and both NR4A3 and ATF3 appear to be cardioprotective: NR4A3 was associated with improvements in infarct size and cardiac function, as well as declines in inflammation when overexpressed in infarcted mouse hearts (Jiang et al., 2019), and measures of cardiac fibrosis, hypertrophy, and function, as well as glucose tolerance, were worse in mice carrying a cardiac-specific ATF3-knockout mutation than in wild type mice when the animals were fed a high-fat diet (Kalfon et al., 2017). There are 38.15% CM1 (Figure 5C) cells co-expressing PRKAG2, NR4A3, and ATF3. Then, multiplying the percentage of PRKAG2 + NR4A3 + ATF3 + CM1 cells by the proportion of CM1 cells (38.15%) in ARP1P28 cardiomyocytes (62.91%) yields 24.00%. This PRKAG2 + NR4A3 + ATF3 + percentage is similar to CM2 percentage in ARP1MIP28P30 and ARP1MIP28P35. Collectively, these observations suggest that CM1 may be composed of the mix-transition states. Also, PRKAG2, NR4A3, and ATF3 may function as molecular switches that trigger the transformation of CM1 cardiomyocytes into CM2 cardiomyocytes. Meanwhile, from the same analysis, transformation from CM1 into CM10 cardiomyocytes may associate with the expressions of RGS6 and TMEM126A, which consistently upregulated in CM1b and CM10.

### CM1 Markers ERBB4 and GRK5 Are Highly Expressed in Regenerative Hearts

**FIGURE 6:**
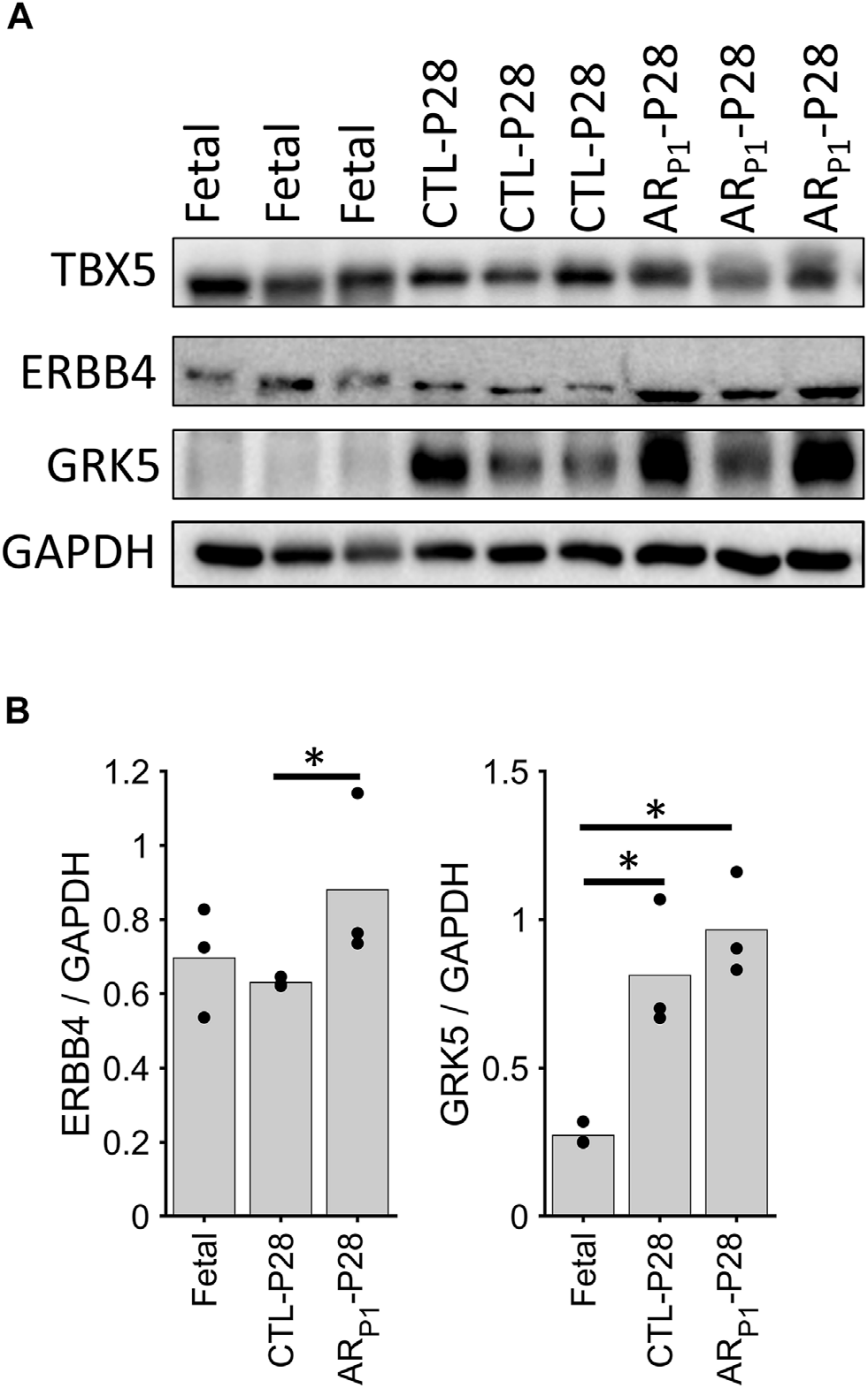
Western blotting confirming ERBB4 and GRK5 expression. The expression in each sample was scaled according to GAAPDH level to ensure equal loading. **(A)** Representative Western Blotting image in each group. Each row presents a protein, in order: ERBB4, and GRK5. Each column presents a sample, in order: Fetal (3 samples), CTL-P28 (3 samples), and AR_P1_-P28 (3 samples). **(B)** Bar chart comparing the average expression among groups. Here, the value for each sample is represented in a circle dot. The horizontal lines and stars (*) represent nonparametric statistical comparisons between two groups. *p-value < 0.05.

The roles of TBX5 (Maitra et al., 2009; Misra et al., 2014), TBX20 (Chakraborty and Yutzey, 2012; Xiang et al., 2016), and ERBB4 (Bersell et al., 2009) in cardiomyocyte proliferation had been reported in previous independent studies; therefore, we focused on validating ERBB4 and GRK5, which localize on the cell surface, protein expression in our pig tissue. The western blotting of ERBB4 and GRK5 showed increased protein levels in AR_P1_-P28, which is consistent with our snRNA-seq data (Figure 6). Western blotting measures the protein expression in the whole tissue, including cardiomyocytes and noncardiomyocytes; therefore, we performed further immunofluorescence validation (cardiomyocyte-specific).

**FIGURE 7:**
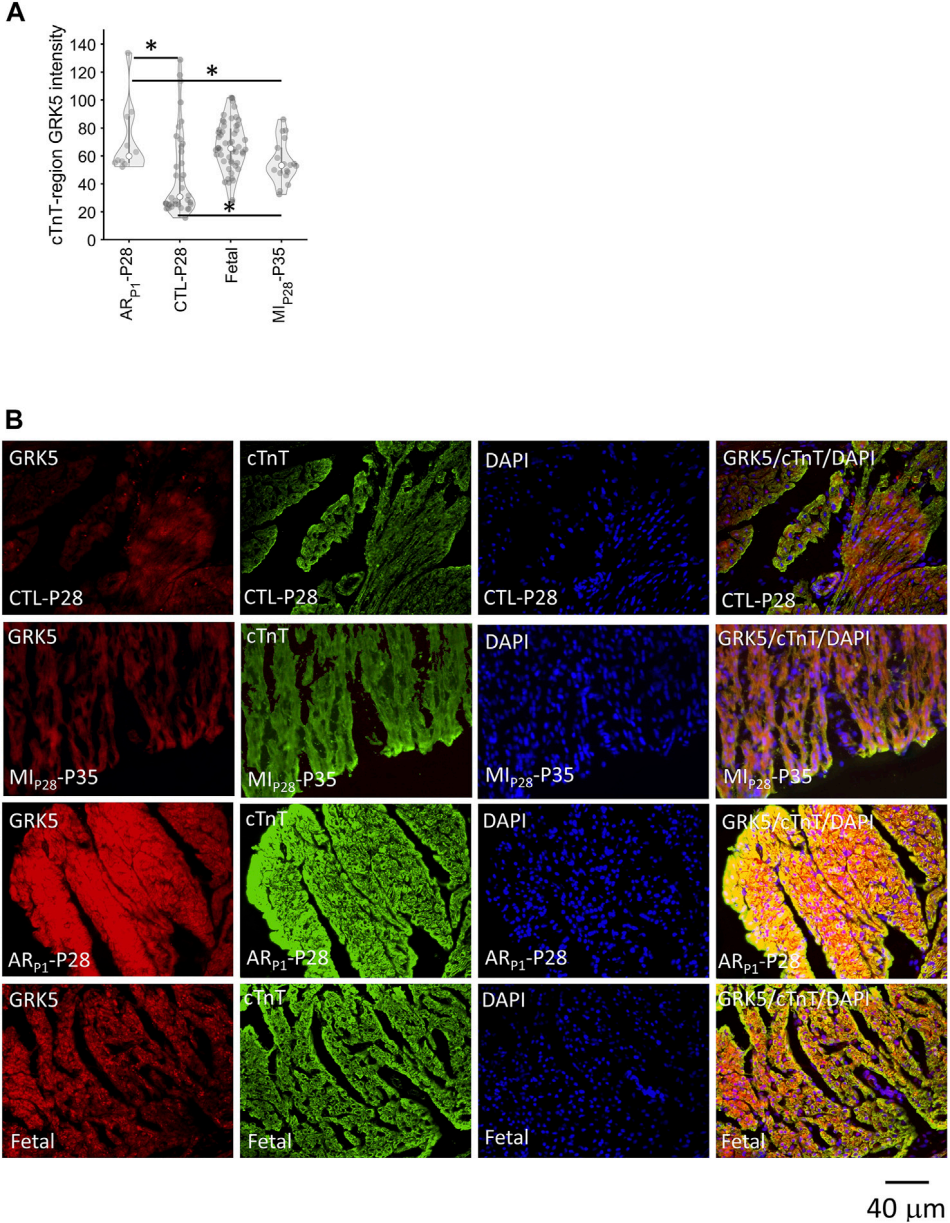
Immunofluorescence analysis confirming GRK5 expression in cardiomyocytes. Here, the expression of GRK5 was represented by the average GRK5 red-channel value (light intensity) that is overlapped with cardiac troponin (cTnT) blue-channel (foreground), which was calculated after adjusting GRK5 red-channel background. DAPI (blue) indicates nuclei. **(A)** Violinplot of GRK5 intensity in 118 staining images. Here, the horizontal bar and the star (*) indicate nonparametric Wilcoxon’s rank sum test between two sample groups. *p-value < 0.05. **(B)** Representative images of GRK5/cTnT/DAPI in each group.

We are interested in investigating the specific expression of GRK5 during heart regeneration as existing GRK5 studies (Martini et al., 2008; Islam et al., 2013; Traynham et al., 2016; de Lucia et al., 2022) have not found the role of GRK5 in cardiomyocyte proliferation. Therefore, additional immunofluorescence analysis (Figure 7) was performed to show the GRK5 expression in cardiomyocytes undergoing the regenerative or non-regenerative process. We found that GRK5 expression increases (*p* = 0.03) 7 days after MIP28 injury in the control (CTL-P28 and MI_P28_-P35) group. But the increased GRK5 level in the control group is still less than its level in AR_P1_-P28 samples (*p* = 0.03). This result is consistent with snRNA-seq analysis, where GRK5 expression is the most elevated in CM1 (exclusively for AR_P1_-P28) among the injured-heart cardiomyocytes. Surprisingly, immunofluorescence analysis shows that GRK5 is also highly expressed in the Fetal group, where cardiomyocytes are expected to actively proliferate.

## Discussion

Although the proliferative capacity of mammalian cardiomyocytes is extremely limited, the meager amount of myocardial regeneration that occurs after MI in the adult heart appears to require the presence of cycling cardiomyocytes (Laflamme and Murry, 2011). A number of studies have shown that mammalian cardiomyocytes remain proliferative for only a few days after birth (Porrello et al., 2011; Lam and Sadek, 2018; Ye et al., 2018; Zhu et al., 2018) but when AR surgery was performed in pigs on P1, cardiomyocytes proliferated in response to MI injury that occurred on P28, and the animals fully recovered with little or no evidence of contractile dysfunction or myocardial scarring (Zhao et al., 2020). The snRNA-seq analyses presented here build upon these previous observations by showing that the proportion of cycling cardiomyocytes increased in response to either AR_P1_ or MI_P28_, and in animals that underwent both surgical procedures, AR_P1_ appeared to further upregulate cardiomyocyte cell-cycle activity for 1 week after MI induction.

Cluster analysis of our snRNA-seq data indicated that cardiomyocytes in the hearts of Fetal pigs, CTL (uninjured) neonatal pigs, and neonatal pigs that underwent AR_P1_, MI_P28_, or both (AR_P1_MI_P28_) can collectively be grouped into 10 different subpopulations (CM1–CM10), and that the abundance of each cluster varied depending on the injury group and time-point analyzed. Cell-cycle gene expression tended to be highest in CM8, which is consistent with our observation that this cluster comprised >99% of the cardiomyocytes in fetal hearts but was absent in all other groups, and cell cycle activity was greater in CM1–7 and CM10 than in CM9, which was almost exclusively associated with CTL-P56 hearts. Notably, the CM1 cluster comprised >60% of the cardiomyocytes in AR_P1_-P28 hearts but was absent in all other groups, including CTL-P28 hearts, which suggests that CM1 cardiomyocytes may have a prominent role in the AR_P1_-induced enhancement of myocardial regeneration. As in Figure 1B, CM1 is very separated from CM8 (representing fetal cardiomyocytes) and CM7 (representing neonatal day 1 cardiomyocyte). Therefore, CM1 is more likely associated with the later postnatal period regeneration rather than the neonatal period. In addition, we hypothesize that CM1 responds to the second myocardial infarction injury on the postnatal day 28 (MI_P28_) by transiting to CM2 or CM10. This response may change the transcription profile of CM1 cardiomyocytes; therefore, the analysis may not represent this “CM1—the same cluster” in AR_P1_-MI_P28_-P30 and AR_P1_-MI_P28_-P30. Therefore, we performed semi-supervised learning to find which regulators might determine the transition from CM1 into CM2 or CM10. Interestingly, proliferative regulators, TBX5 and GRK5 continued expressing highly in CM2. Different from our previous publication (Nakada et al., 2022), which only detected six cardiomyocyte subpopulations, the newly added data, and AI-based pipeline enabled significantly deeper analysis, resulting in ten subpopulations. Beyond reconfirming the PKM2+/NEB + subpopulation as in (Nakada et al., 2022), this work characterized four cardiomyocyte subpopulations in injured-heart cardiomyocytes (CM1, CM2, CM10, and CM6). Furthermore, transitions among these subpopulations (CM1, CM2, and CM10) were investigated, which had not been examined in our previous work (Nakada et al., 2022).

Cell-cycle activity was not substantially greater in CM1 cardiomyocytes than in cardiomyocytes from any other cluster except CM9. However, TBX5, TBX20, ERBB4, and GRK5, which have been linked to the proliferation of fetal and neonatal cardiomyocytes [TBX5 (Misra et al., 2014; Maitra et al., 2009), TBX20 (Xiang et al., 2016; Chakraborty and Yutzey, 2012), ERBB4 (Bersell et al., 2009)] and cardiac hypertrophy [GRK5 (Gold et al., 2012; Traynham et al., 2015)], were upregulated in the CM1 cluster, as well as in Fetal cardiomyocytes. Bioinformatics analysis (Figure 3B) showed that TBX5 and TBX20 transcription factors amplified the expression of positive regulation of cell proliferation, cardiac muscle cell differentiation, canonical Wnt signaling pathway, and MAPK signaling pathway makers. Furthermore, ERBB4 and GRK5, which are surface receptor proteins, may activate ERBB, canonical Wnt, and MAPK signaling pathways at downstream. Thus, AR on P1 may have primed cardiomyocytes for cell-cycle reactivation by maintaining the signaling mechanisms associated with these four molecules through at least P28. TBX5 and GRK5 were also highly expressed in CM2 cardiomyocytes, which comprised ∼20% of cardiomyocytes in AR_P1_-P28 hearts and a substantially greater proportion of cardiomyocytes in AR_P1_MI_P28_ than in MI_P28_ hearts during the first week after MI induction. This 7-day window coincided with the period when cycling cardiomyocytes were significantly more common in AR_P1_MI_P28_ hearts, and although we did not observe differences between cardiomyocyte clusters, injury groups, or time-points in the expression of downstream effectors of these signaling pathways, the results from bulk RNA sequencing assessments suggested that MI induction on P1 upregulated the expression of two molecules identified in our network analysis, MEF2C and NKX2-5, as well as TBX5 (Zhang et al., 2020). CM1 and CM2 cardiomyocytes also displayed substantial evidence of both glycolytic and βFAOX metabolism, while most of the other cardiomyocytes (i.e., CM10 and CM4) in AR_P1_MI_P28_ and MI_P28_ hearts on P30 and P35 relied primarily on glycolysis for energy production, but whether TBX5 or GRK5 activate βFAOX in neonatal cardiomyocytes, and if so, whether βFAOX upregulation improves the regenerative response to myocardial injury, has yet to be investigated.

The TBX5+/TBX20+/ERBB4+/GRK5+ cardiomyocyte subpopulation (CM1) and its markers were not detected in our previous report (Nakada et al., 2022). This subpopulation was detected in this study by adding new snRNA-seq data and applying the artificial-intelligence techniques. Pipelines to analyze snRNA-seq data include unsupervised clustering (Kiselev et al., 2019), which means the technical result depends on the data being used. Given the same data, it is known that different pipelines can lead to different results (Vieth et al., 2019). Therefore, whether the novel CM1 subpopulation appeared in this study primarily due to adding new data or using a different pipeline is yet to be investigated. From the computational perspective, compared to the Seurat-based pipeline (Hao et al., 2021) used in (Nakada et al., 2022), the artificial-intelligence-based pipeline gives the dimensional reduction advantage. Before clustering, artificial intelligence (autoencoder) reduced the high-dimensional snRNA-seq data into 10 dimensions; meanwhile, Seurat, applying Principal Component Analysis still reduced into 2,000 dimensions. Notably, both of them use the same principle to compute lower-number dimensions: when using the lower-number dimensions to reconstruct the data, the difference between the reconstructed data and original high-dimensional data is minimized. In computing, given the similar optimization criteria, using a lower number of dimensions is better to “curse of dimensionality” issue (Trunk, 1979): the calculation is much less accurate if too many data dimensions are used.

As support of our finding, the roles of TBX5, TBX20, and ERBB4 in promoting cardiomyocyte proliferation had been reported in other studies. In addition, we confirmed the evaluated protein levels of ERBB4 and GRK5 in regenerative heart tissue. Therefore, immunofluorescence analysis was performed to quantify GRK5 expression within cardiomyocytes, where we confirmed the signal intensity of the cTnT region/GRK5 in AR_P1_-P28 and Fetal groups were higher. Overall, our results suggest that GRK5 may contribute to cardiomyocyte proliferation, whose mechanisms are yet to be further confirmed in future studies.

In conclusion, the results presented here identified a cluster of cardiomyocytes, CM1, in neonatal pig hearts that appeared to be generated in response to AR injury on P1, which in turn, results in CMs cell-cycle activation, and has improved recovery from subsequent AMI on P28. Although CM1 cardiomyocytes did not appear to be substantially more proliferative than cardiomyocytes from other clusters that were present in injured and uninjured hearts through P42, they may have been primed for cell-cycle reactivation by the upregulation of regulatory molecules that contribute to cardiomyocyte proliferation (TBX5, TBX20, and ERBB4) and cardiac hypertrophy (GRK5). Collectively, these observations support future investigations of the roles of these four regulatory molecules in cardiomyocyte proliferation and myocardial repair.

## Materials and Methods

### Animals

All experimental protocols were approved by The Institutional Animal Care and Use Committee (IACUC) of the University of Alabama, Birmingham, and performed in accordance with the National Institutes of Health Guide for the Care and Use of Laboratory Animals (NIH publication No 85-23). Pigs were purchased from Prestage Farm, Inc. (West Point, MS) and cared for as described previously (Zhao et al., 2020). Pigs were housed in an incubator at ∼85°F with room air through P14 and then transferred to the normal room and grown under regular pig feeding and ∼72°F temperature. Animals were fed bovine colostrum every 4 h for the first 2 days of life, a 1:1 mixture of colostrum: sow’s milk on day 3 of life, and sow’s milk thereafter. Supplemental iron was provided on day 7.

### Aapical Resection and Myocardial Infarction–Induction Surgery

Pigs were anesthetized with isoflurane and placed on a heating pad in a dorsal recumbent position; then, a median sternotomy was performed to expose the heart. AR was performed on P1 by removing 4–5 mm of tissue from the ventricular apex, and MI was induced on P28 by permanently occluding the left-anterior descending coronary artery with a suture; then, the sternum was reapproximated, and the chest was closed in layers, and the air was evacuated from the mediastinum.

### Nuclei Isolation

Tissues were cut while submerged in cold phosphate-buffered saline (PBS) or UW solution, washed to remove blood, and transferred into 1 ml lysis buffer (10 mM Tris-HCl, 10 mM NaCl, 3 mM MgCl_2_, 0.1% Nonidet™ P40 Substitute, and 50 U/ml RNase inhibitor in DEPC-treated water), cut into smaller pieces, and aspirated with the lysis buffer into a 50-ml tube; then, 10 ml of lysis buffer was added, the tissues were ground for 20–30 s, and more lysis buffer was added. The mixture was placed on ice for 10 min, filtered with 100-μm and 70-μm strainers, and centrifuged for 5 min at 700 rcf and 4°C; then, the supernatant was removed, and the pellet was resuspended in 10 ml nuclei wash and resuspension buffer [1 × PBS, 1.0% bovine serum albumin (BSA), and 50 U/ml RNase inhibitor]. The suspension was passed through a 40-μm strainer, and the nuclei were centrifuged again for 5 min at 700 rcf and 4°C; then, the supernatant was removed, the pellet was resuspended in 1 ml nuclei wash, and resuspension buffer and the nuclei were centrifuged a third time for 5 min at 700 rcf and 4°C. The supernatant was removed; then, the pellet was resuspended in 5 ml sucrose cushion buffer I (2.7 ml Nuclei PURE 2M Sucrose Cushion Solution and 300 μl Nuclei PURE Sucrose Cushion Buffer) and mixed with 10 ml sucrose buffer. The mixture was layered over 5 ml of sucrose cushion buffer in a second Eppendorf tube and then centrifuged for 60 min at 13,000 g and 4°C. All but 100 μl of the supernatant was removed, the nuclei were resuspended in nuclei wash and resuspension buffer, and the solution was passed through a 40-μm strainer; then, the nuclei concentration was determined with a cell counter or hemocytometer and adjusted to 1,000 nuclei/μl. The nuclei were placed on ice, stained with propidium iodide for 5 min, and then immediately processed *via* the 10× Genomics^®^ Single-Cell Protocol.

### Pre-Processing of snRNA-Seq Data

Sample demultiplexing, barcode processing, and gene counting were performed with Cell Ranger Single-Cell Software v.3.10 (https://support.10xgenomics.com/single-cell-gene expression/software). Reads were aligned to the Sscrofa11.1 pre-mRNA reference genome (Pig, 2021), and the pre-mRNA portion of the reference genome was extracted for single-nuclei UMI mapping with Cell Ranger mkref pre-mRNA. Only confidently mapped reads with valid barcodes and UMIs were used to generate the gene-barcode matrix. Cross-sample integration and quality-control were performed with the Seurat R package. Doublets were identified by using Seurat’s standard pipeline (https://satijalab.org/seurat/v3.2/pbmc3k_tutorial.html), and barcodes were removed if they had fewer than 500 UMIs, more than 30,000 UMIs, or >5% mitochondrial UMIs. Nuclei were removed if they had <200 detected genes or if >25% of the transcripts were from mitochondrial genes. Mitochondrial genes and other transcript identifiers were removed without mapping to the official gene symbols from later analyses. Data were normalized as directed in the online Seurat tutorial (https://satijalab.org/seurat/v3.2/pbmc3k_tutorial.html); total expression was multiplied by a factor of 10,000 and then log-transformed. Variations in the number of genes and UMIs detected per cell were accommodated by normalizing the scaled expression matrix *via* the ScaleData function with vars. to. regress set to nUMI and nGenes. Normalization returned two gene-cell matrices: the first was in log scale, and the second was the adjusted gene-cell count.

### Autoencoder

An autoencoder (Kramer, 1991) is a deep-neural-network artificial-intelligence technique used to synthesize (Sheikh et al., 2021), denoise (Eraslan et al., 2019), or translate (Cho et al., 2014) data. A data-synthesis autoencoder has at least three layers: an input layer, which consists of the original high-dimensional dataset, a central embedded layer with fewer dimensions (typically 10–20), and a synthetic output layer whose dimensionality is equivalent to the input layer. The autoencoding procedure is performed by encoding the input layer into the embedded layer, decoding the embedded layer into the output layer, and then evaluating the extent of similarity between the input and output layers. When the output layer matches the input layer with maximum fidelity, the embedded layer is considered an accurate low-dimensional representation of the input data. Notably, the immense datasets generated *via* single-cell and single-nucleus genomic analyses can require a prohibitively large amount of computer memory, so a number of recent studies (Wang and Gu, 2018; Eraslan et al., 2019; Tran et al., 2021) have reduced the dimensionality of the input data before the autoencoding procedure is initiated. However, the transcriptional heterogeneity of cardiac cells is exceptionally high (Nahrendorf and Swirski, 2013; Vidal et al., 2019; Tsedeke et al., 2021) and likely to be exacerbated by the physiological changes that occur in response to AR surgery and MI induction. Thus, since any attempt to reduce the dimensionality of the input data before autoencoding could mask this complexity, the input layer used for the studies reported here included the complete list of 14,753 genes (i.e., 14,753 dimensions) with at least 1,000 UMIs in our dataset.

Autoencoding was performed in Matlab (trainAutoencoder, 2021) with self-built models; the architecture of the autoencoder was restricted to three layers, and the embedded layer contained 10 dimensions (Supplementary Figure S5). Optimization was achieved by minimizing the function

$$E=\frac{1}{N}\overset{N}{\underset{i}{\sum }}\overset{N}{\underset{j}{\sum }}{\left(x_{i}-y_{j}\right)}^{2}+0.001||W{||}^{2}+Q,$$ (1)

where *N* denotes the number of data points, *x*_*i*_ denotes an arbitrary input data point, *y*_*j*_ denotes an arbitrary synthetic data point, ||*W*||^2^ represents the regularization of autoencoder weights, and *Q* represents the sparsity parameters (trainAutoencoder, 2021).

### Data Visualization and Clustering

After autoencoding, the embedded data was reduced from 10 to 2 dimensions *via* Uniform Manifold Approximation (UMAP) toolkit (McInnes et al., 2018; Meehan, 2021), and clustering was performed with the UMAP toolkit density-based clustering (dbscan) algorithm (McInnes et al., 2018; Meehan, 2021; Ester et al., 1996; dbscan, 2021); the 30th-distance graph (minpts, or n_neighbors = 30) was plotted, and epsilon (or min_dist) was set to 0.3 (Meehan, 2021). Cardiomyocytes were identified *via* expression of the cardiomyocyte-specific markers ACTC1 and MYH7 (Supplementary Note S1); then, the cardiomyocyte data was extracted, and the autoencoding, visualization (UMAP), and clustering (dbscan) procedures were repeated. The assignment of cardiomyocyte clusters (CM1–CM10) was based on visualization and the distribution of cardiomyocytes in each subgroup (Supplementary Note S2), and marker genes for each cluster were identified according to the following criteria: 1) a cluster *p*-value [Fisher’s Exact test (Fisher, 1922)] of less than 10^−6^, 2) expression by at least 50% of cells in the cluster, and 3) mean abundance at least 1.3-fold greater among cells in the cluster than in the total cardiomyocyte population.

### Algorithm Quality Analysis

After being clustered UMAP toolkit (McInnes et al., 2018; Meehan, 2021), the snRNA-seq clusters were validated and manually adjusted (if needed) by the cell-groups’ localization in each sample group. In Supplementary Figure S6, the cell-landscape in each group revealed specific regions that were significantly enriched by or were exclusive for AR_P1_-P28, AR_P1_-MI_P28_-P30/P35, injured P56, Fetal, CTL-P1, and CTL-P56 cardiomyocytes. There were regions mapped to CM1, CM2, CM6, CM8, CM7, and CM5/CM9, respectively (Supplementary Figure S7B). This result explained and justified our clustering parameter settings. This strategy was also used in other single-cell analyses, such as in (Cui et al., 2020).

In addition to the (minpts = 30, epsilon = 0.3) parameter, combination was manually examined according to the instruction in (DBSCAN, 2022). Briefly, for each cell-point, the distances between the point and 30 other nearest points were calculated; then, among these 30 distances, the largest distance was selected as “30th nearest distance.” Repeating this process for all cell-points, the list of “30th nearest distances” for all cell-points between 0.1 and 1.2 was obtained, as displayed on the *y*-axis of Supplementary Figure S7A. At any threshold *t* on the *y*-axis, the number of cell-points *m* having “30th nearest distances” < *t* was counted, and the (*m*, *t*) dot was drawn in Supplementary Figure S7A. Repeating the (*m*, *t*) process as t increased from 0.1 to 1.2 and connecting these (*m*, *t*) dots, the “30th nearest distance curve” was completed as shown in Supplementary Figure S7A. On “30th nearest distance curve,” “the elbow” (*m*, *t*) point corresponds to *t* ≈ 0.3. This result further justified our decision to use the parameter combination (minpts = 30, epsilon = 0.3).

On the other hand, we manually examined the clustering result when slightly changing minpts = 25, 30, and 35, and epsilon = 0.25, 0.3, and 0.35 with the Matlab UMAP toolkit (Meehan, 2021). Changing these parameters may yield different numbers of clusters. However, using the cell groups localization to manually merge the smaller clusters, we reconstructed a nearly identical cluster landscape to what was obtained using (minpts = 30, epsilon = 0.3) parameter combination. This result justified the robustness of the cluster algorithm and toolkit.

We also justified the criteria by the number of marker genes and their percentage over the entire pig genome, which consists of 25,800 genes. Table 1 shows that the number of genes passing two criteria (to qualify as cluster markers) is always less than 2% of the genome. Furthermore, we combined the marker genes from all 10 CM clusters, yielding 1,069 marker genes. Then, for the gene, we counted in how many CM clusters such that the gene was a marker. There were 636 genes (59.50%) that were markers of only one CM cluster; 312 genes (29.19%) that were markers of two CM clusters; 98 genes (9.17%) that were markers of three clusters; 21 genes (1.96%) that were markers of four clusters; and only 2 genes (0.19%) that were markers of five clusters. There were no genes that were markers of six or more clusters. Therefore, the criteria to select cluster markers ensured that the markers were very specific for the cluster.

**TABLE 1:** Number and proportion of genes passing the cluster marker criteria: expressing in more than 50% of the cluster cell and having at least 1.3-fold abundance.

Cardiomyocyte (CM) cluster	Criterion 1: genes expressed in more than 50% of the cluster cell		Criterion 2: expression is 1.3-fold higher (than other clusters)		Criterion 1 and Criterion 2	
	#Genes	%Genome (%)	#Genes	%Genome (%)	#Genes	%Genome (%)
CM1	881	3.40	2,263	8.74	126	0.49
CM2	964	3.73	1,221	4.72	74	0.29
CM3	1,541	5.95	5,610	21.68	419	1.62
CM4	1,435	5.54	9,670	37.36	274	1.06
CM5	1,090	4.21	1,935	7.48	109	0.42
CM6	1,073	4.15	1,209	4.67	89	0.34
CM7	862	3.33	4,438	17.15	102	0.39
CM8	807	3.12	6,544	25.29	206	0.80
CM9	855	3.30	1,277	4.93	241	0.93
CM10	912	3.52	1,391	5.38	8	0.03

### DAVID Functional Annotation

Cluster-specific markers were analyzed with the DAVID functional annotation tool (Huang et al., 2009). Only terms present in the manually annotated Gene Ontology (Huntley et al., 2015), KEGG (Kanehisa et al., 2017), and Reactome (Jassal et al., 2020) categories were selected. To avoid false discoveries caused by multi-hypothesis testing, the selected results were required to have *p* < 0.01 or Benjamini-adjusted (Li and Barber, 2019) *p* < 0.05.

### Sparse Modeling

Because the markers selected *via* our DAVID functional analysis were restricted to those present in manually annotated databases, complementary analyses were conducted without the selected markers *via* Sparse modeling. Briefly, for each cell expression data vector **x**, the sparse model estimates a score *y* from the linear formula

y=wx+b. (2)

Only genes known to be associated with the ontology or pathway being evaluated were considered [e.g., G2M scores were calculated using only genes with known G2M ontology (Mouse Genome Informatics, 2021a)], and higher *y* implied that the cell was more likely to undergo the corresponding process. Sparse analysis also requires predefined “positive” (*y* = 1) and “negative” (*y* = –1) cell groups (Table 2); thus, cell-cycle gene scores (for example) were calculated with Fetal and CTL-P56 cardiomyocytes as the positive and negative groups, respectively, because Fetal cardiomyocytes are highly proliferative, whereas CTL-P56 cardiomyocytes have exited the cell cycle.

**TABLE 2:** Positive and negative cell groups for Sparse analysis.

Analysis	Positive cell group	Negative cell group
Cell cycle phases (Cui et al., 2020) (G01, G1S, S, G2M, M, and MG1)	Fetal cardiomyocytes	Naïve-P56 cardiomyocytes
Cardiomyocyte adrenergic signaling (KEGG: Adrenergic signaling in cardiomyocytes, 2021)	CM9*	CM8*
Cardiac muscle contraction (Mouse Genome Informatics, 2021b)	CM9	CM8
Cardiac muscle cell development (Gene Ontology: Cardiac Muscle Cell Development, 2021)	CM8	CM9
Positive regulation of cardiac muscle cell proliferation (Mouse Genome Informatics, 2021c)	CM8	CM9
Positive regulation of cardiac muscle tissue growth (Mouse Genome Informatics, 2021d)	CM8	CM9
Glycolysis (Glycolysis, 2021)	CM8	CM9
Beta fatty acid oxidation (Mouse Genomic Informatics, 2021b)	CM9	CM8

*The CM8 and CM9 clusters were exclusively associated with cardiomyocytes from Fetal and CTL-P56 hearts, respectively.

The **w** and *b* (bias) parameters of Eq. 1 were computed by minimizing

$$\frac{1}{2}\left|w\right|+C\underset{\forall i}{\sum }\epsilon_{i}.$$ (3)

Subject to

$$\{\begin{array}{c} y_{i}\left(wx_{i}+b\right)+\epsilon_{i}\geq 1\\ \epsilon_{i}\geq 0 \end{array}\;\;\forall i,$$ (4)

where *i* denotes an arbitrary “positive” or “negative” cell and the “margins” of the model are defined by wx+b =1 and wx+b =−1 . A good model will have a very high percentage (e.g., 90%) of cells with $\epsilon_{i}=0$ .

Scores were computed for all cells; then, cells with *y* > 1 (from Eq. 2) were categorized “high,” cells with *y* < –1 were categorized “low,” and cells with –1 ≤ *y* ≤ 1 were categorized “middle.” Thus, a “high” G2M categorization (for example) indicated that the cell was more Fetal- than CTL-P56–like and, consequently, more likely to execute the G2M phase transition. Significance was evaluated by calculating the OR and *p*-value [Fisher’s Exact Test (Fisher, 1922)] for the proportion of “high” cells in each group, and *p* < 10^−6^ was considered significant.

### Network Analysis for TBX5, TBX20, ERBB4, and GRK5

Genes targeted by TBX5 and TBX20 were identified in the TRRUST v2 database (Han et al., 2018), and genes that function downstream of ERBB4 and GRK5 were identified in the STRING v11.5 (Szklarczyk et al., 2021) database. Identified genes were combined into a single set and analyzed *via* DAVID (Huang et al., 2009).

### Semisupervised Learning Analysis

The semisupervised learning model was built with the Matlab “fitsemiself” function (Matlab, 2021); CM2 cardiomyocytes from AR_P1_MI_P28_-P30 hearts were used as the class 1 cells, and CM10 cardiomyocytes from AR_P1_MI_P28_-P30 hearts were used as the class 2 cells. For each CM1 cardiomyocyte from AR_P1_-P28 hearts, the model calculated a rescaled score between 0 and 1; cells with scores approaching 0 or 1 were categorized an CM1a (i.e., CM2-like) or CM1b (i.e., CM10-like), respectively.

### Cardiomyocyte Pairwise Cluster Similarity Analysis

The similarity between two cardiomyocyte clusters, denoted as CM*x* and CM*y*, was calculated by averaging 1,000 similarity scores between 1,000 randomly selected cells in CM*x* and 1,000 randomly selected cells in CM*y*. The cell-cell similarity was computed using the following equation

$$\sqrt{\overset{m}{\underset{j=1}{\sum }}{\left(sign\left(x_{j}\right)-sign\left(y_{j}\right)\right)}^{2}}.$$ (5)

Here, *x,y* represents the gene expression of a randomly selected cell in CMx and CMy, correspondingly, *m* is the number of genes, and *j* represents the gene index. The “sign” function converts the expression into the binary (0 or 1) format, whereas *x*(*i*) = 1 means the *i*th gene expresses in cell *x*, while *x*(*i*) = 0 means the *i*th gene does not express in cell *x*.

### Bulk-RNA Sequencing Analysis

The regenerative pig heart bulk-RNA sequencing data was obtained from the Gene Expression Omnibus database, accession number GSE144883, and processed according to (Zhang et al., 2020). Briefly, the paired-end fastq files were trimmed using TrimGalore (Krueger, 2019), then mapped to Ensembl Sscrofa11.1 pre-mRNA reference pig genome (Pig, 2021) using STAR v.2.5.2 (Dobin et al., 2013), then the transcripts for each gene (raw expression) were counted using HtSeq v.0.6.1 (Anders et al., 2015). Then, the raw expression matrix was normalized using Deseq2 (Love et al., 2014). Expressions between 3 regenerative hearts, which underwent myocardial infraction on postnatal day 1, and 3 naïve hearts were compared, and error-bar plotted.

### Western Blotting

The Western blotting protocol, which quantified protein expression of ERBB4, GRK5, and GAPDH was completed according to our previous publication (Zhang et al., 2020). Tissues were lysed in PIPA Lysis and Extraction Buffer (Thermo Scientific, 89,901) with Halt™ Protease and phosphatase Single-Use Inhibitor Cocktail (Thermo Scientific, 78,442); then, the lysates were denatured at 95°C for 6°min, separated in a 4%–20% precast gel (Bio-rad, 4568093), and transferred onto a PVDF membrane (Bio-rad, 1704156). The membrane was incubated with 5% nonfat dry milk (Bio-rad, 1706404) for 30 min, with primary antibodies at 4°C overnight, and then with horseradish-peroxidase (HRP)–conjugated secondary antibodies for 30 min. Protein bands were detected with the chemiluminescent HRP substrate (Millipore, WBKLS0500) in a ChemiDocTM Imaging System (Bio-rad).

We performed western blotting in three Fetal, AR_P1_-P28, and three CTL-P28 samples. In each sample, protein expression was scaled according to GAPDH to confirm equal loading. Then, statistical comparison and testing were performed using the nonparametric test, according to (Zhao et al., 2021), due to the small sample size. A *p*-value < 0.05 indicates statistical significance.

### Histology

The immunofluorescence analyses were conducted similar to our previous work in Nakada et al. (2022). Hearts were cut into transverse blocks (thickness: 1 cm), and myocardium from the anterior-apical zone (AAZ) were either snap-frozen with liquid nitrogen or processed with 10% formalin fixation and dehydration with 10%- 30% sucrose overnight. Samples were cut into transverse sections (thickness: 10 μm) and stained with antibodies against GRK5 (1:100, rabbit polyclonal, Invitrogen, PA5-106484) and cardiac troponin T (1:50, mouse monoclonal, R&D System, MAB 1874) overnight was followed by blocking in Ultravision Protein Block (Epredia, TA125PBQ) for 7 min. For each group, at least 10 sections of border zone myocardium were analyzed, and a total of 118 images from subendomyocardium, and subepimyocardium were counted. Anti-rabbit and anti-mouse secondary antibodies conjugated to Alexa Fluor 555 and 488 were used for visualization by microscopy. DAPI was used for nuclei staining.

To quantify the GRK5 light intensity at the cardiac troponin T area, the following image processing pipeline was performed (Supplementary Figure S8). First, each staining image (including GRK5 and cTnT) was represented in Matlab by the Red-Green-Blue channel matrices. Here, in the red-channel matrix, the number between 0 (totally no red) and 255 (maximum red) represents the red color intensity, so does in the green-channel and blue-channel. Then, image segmentation was performed in the cTnT image green channel: green >10 implied the “foreground” (with cTnT) areas, while green <10 implied the “background” (Supplementary Figure S8B). Then, the “segmented” image was mapped to the GRK5 red channel, where this channel was adjusted by subtracting with the background red channel baseline (Supplementary Figure S8C). GRK5 intensity was calculated by the mean of the foreground red-channel (adjusted) number, which was between 0 and 255. Since only the overlap between GRK5 (red) and cTnT (green) areas was used in GRK5 intensity calculation, this approach was better to quantify GRK5 in cardiomyocytes. Then, statistical comparison among the groups was completed using nonparametric Wilcoxon’s Rank sum test. A *p*-value < 0.05 indicates statistical significance.

The background red channel baseline was determined in each GRK5 image based on the distribution (histogram) of the background red channel numbers. Manually investigating all 118 images, we noted three background scenarios. First, when the background numbers follow a power-law distribution, where most of the numbers were around 0 and much fewer nonzero backgrounds, the baseline was set to 0, implying no background adjustment was needed. Second, when the background number appeared to be in a homogeneous distribution, which was either normal, uniform, or power-law, the baseline was set to be the average of the background number. Third, when the background numbers formed two or more distributions, we separated these distributions and set the baseline to be the average of the largest (most right) distribution.

## Data Availability

The datasets presented in this study can be found in online repositories. The names of the repository/repositories and accession number(s) can be found below: https://www.ncbi.nlm.nih.gov/geo/, GSE185289.

## Supplementary Material

Supplementary Figure S1 Cluster analysis identified six different cell types in hearts from Fetal, CTL, ARP1, MIP28, and ARP1MIP28 animals. snRNA-seq data for all cells from each experimental group and time point were processed via cluster analysis and visualized *via* UMAP. **(A)** Results are displayed for each experimental group and time point. **(B)** Results are displayed for each of six different cell types (cardiomyocytes, fibroblasts, immune cells, smooth muscle cells, endothelial cells, and cardiomyocyte/skeletal-like muscle cells). **(C)** The expression of cell-type–specific genes that were explicitly associated with each cluster is displayed as a heat map (cardiomyocytes: myosin heavy chain 7 [MYH7], Actin Alpha Cardiac Muscle 1 [ACTC1]; fibroblasts: Collagen Type I Alpha 1 [COL1A1], Collagen Type I Alpha 2 [COL1A2]; immune cells: Bridging Integrator 2 [BIN2], gamma-interferon-inducible lysosomal thiol reductase [IFI30]; smooth-muscle cells: Actin Alpha 2 [ACTA2], Myosin Heavy Chain 11 [MYH11]; endothelial cells: Platelet And Endothelial Cell Adhesion Molecule 1 [PECAM1], Kinase Insert Domain Receptor [KDR]; skeletal-muscle cells: Myosin Light Chain Kinase 2 [MYLK2], Nebulin [NEB]).

Supplementary Figure S2 Upregulation of pluripotent genes in CM4 cardiomyocyte. **(A)** Regulators in KEGG signaling pathways regulating pluripotency of stem cells, including AKT Serine/Threonine Kinase 3 [AKT3], Janus Kinase 1 [JAK1], Janus Kinase 2 [JAK2], Jumonji And AT-Rich Interaction Domain Containing 2 [JARID2], Mitogen-Activated Protein Kinase 1 [MAPK1], Phosphatidylinositol-4,5-Bisphosphate 3-Kinase Catalytic Subunit Alpha [PIK3CA], Signal Transducer And Activator Of Transcription 3 [STAT3], and Zinc Finger Homeobox 3 [ZFHX3]. **(B)** Genes responsible for stem cell population maintenance according to Gene Ontology annotation, including APC Regulator Of WNT Signaling Pathway [APC], AT-Rich Interaction Domain 1A [ARID1A], DEAD-Box Helicase 6 [DDX6], Forkhead Box O1 [FOXO1], Nuclear Receptor Coactivator 3 [NCOA3], Recombination Signal Binding Protein For Immunoglobulin Kappa J Region [RBPJ], SWI/SNF Related, Matrix Associated, Actin Dependent Regulator Of Chromatin, Subfamily A, Member 2 [SMARCA2], SWI/SNF Related, Matrix Associated, Actin Dependent Regulator Of Chromatin Subfamily C Member 1 [SMARCC1], SS18 Subunit Of BAF Chromatin Remodeling Complex [SS18], and Transcription Factor 7 Like 2 [TCF7L2]. The expression was normalized according to the Seurat pipeline: the expression matrix was scaled by the ScaleData function with vars.to.regress set to nUMI and nGenes; then, the scaled expression was log-normalized.

Supplementary Figure S3 Pairwise similarity among cardiomyocyte CM1-CM10 clusters. The cluster similarity was calculated by averaging 1000 cell-cell similarity scores, whereas the cells were randomly selected in each cluster. The left-side bar maps the color to the similarity score, where more red implies a higher degree of similarity.

Supplementary Figure S4 Cardiomyocyte cell-cycle gene expression tended to be greater in ARP1, MIP28, and ARP1MIP28 hearts from P30-P42 than in CTL hearts on P28. Sparse analysis was conducted for the expression of genes associated with the G01, G1S, S, G2M, M, and MG1 phases of the cell cycle, and the proportion of cardiomyocytes with high levels of expression was calculated for each experimental group and time point.

Supplementary Figure S5 Schematic representation of the autoencoder model. The autoencoder constructed for the analyses presented here consisted of an input layer, a central embedded layer, and an output layer. The input layer included 14,753 genes and was alternately encoded into a 10-dimensional embedded layer and then decoded into a synthetic output layer comprising the same genes present in the input layer. The accuracy of the embedded layer was optimized by minimizing the indicated function.

Supplementary Figure S6 Cell localization in each sample group’s cardiomyocyte. Here, the umap plots for all cells were drawn, and the cells were colored based on: whether the cell belongs to a specific sample group (red) or the cell belongs to other sample groups. The order, from left to right and from top to bottom, is: CTL-P28, ARP1-P28, MIP28-P30, ARP1-MIP28-P30, MIP28-P35, ARP1-MIP28-P35, MIP28-P42, ARP1-MIP28-P42, MIP28-P56, ARP1-MIP28-P56, Fetal, CTL-P1, CTL-P56, and ARP1-P56.

Supplementary Figure S7 Algorithm-quality analysis confirming that the parameter combination (minpts = 30, epsilon = 0.3) in UMAP toolkit is robust. **(A)** The 30th-neartest-distance plot show that the “elbow” point, which determine the optimal epsilon parameter (according to [92]) is approximately 0.3. **(B)** The preserved cluster landscape draw by slightly changing the parameters: minpts = 25, 30, 35, and epsilon = 0.25, 0.3, 0.35.

Supplementary Figure S8 Summary of Immunofluorescence image processing. **(A)** The staining images (red: GRK5, green: cardiac troponin cTnT) were represented in red(R)/green(G)/blue(B) channel matrices. **(B)** Image segmentation was performed on the blue channel, which divided the image into the background (no cTnT, black) and foreground (with cTnT, white). **(C)** The red channel background distribution was plotted to determine a background baseline. This distribution had three scenarios. Top: near-zero distribution, the baseline (indicated by the dashed line) = 0; middle: one distribution form, the baseline = average of red channel background; bottom: the two or more distributions appeared, then the baseline = average of the largest (most right) distribution. **(D)** the red channel (GRK5) was adjusted by subtracting this channel from the background baseline.
