# Supplementary figures and images for: Cardiomyocyte Cell-Cycle Regulation in Neonatal Large Mammals: Single Nucleus RNA-Sequencing Data Analysis via an Artificial-Intelligence–Based Pipeline
Source: Front Bioeng Biotechnol. 2022 Jul 4;10:914450. doi: 10.3389/fbioe.2022.914450 (PMC9289371; doi:10.3389/fbioe.2022.914450)

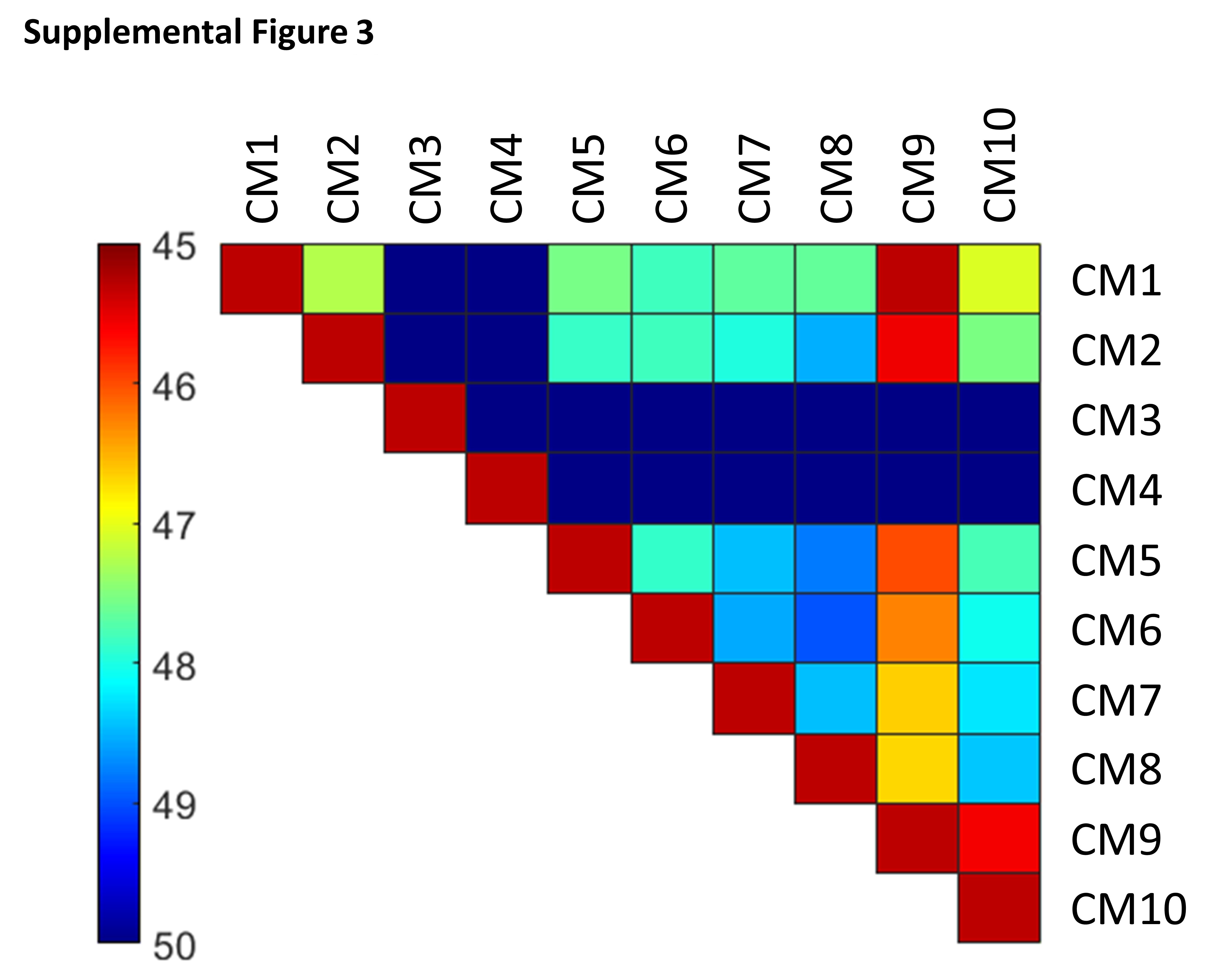

Supplement: Supplementary file 1 [file Image3.JPEG]

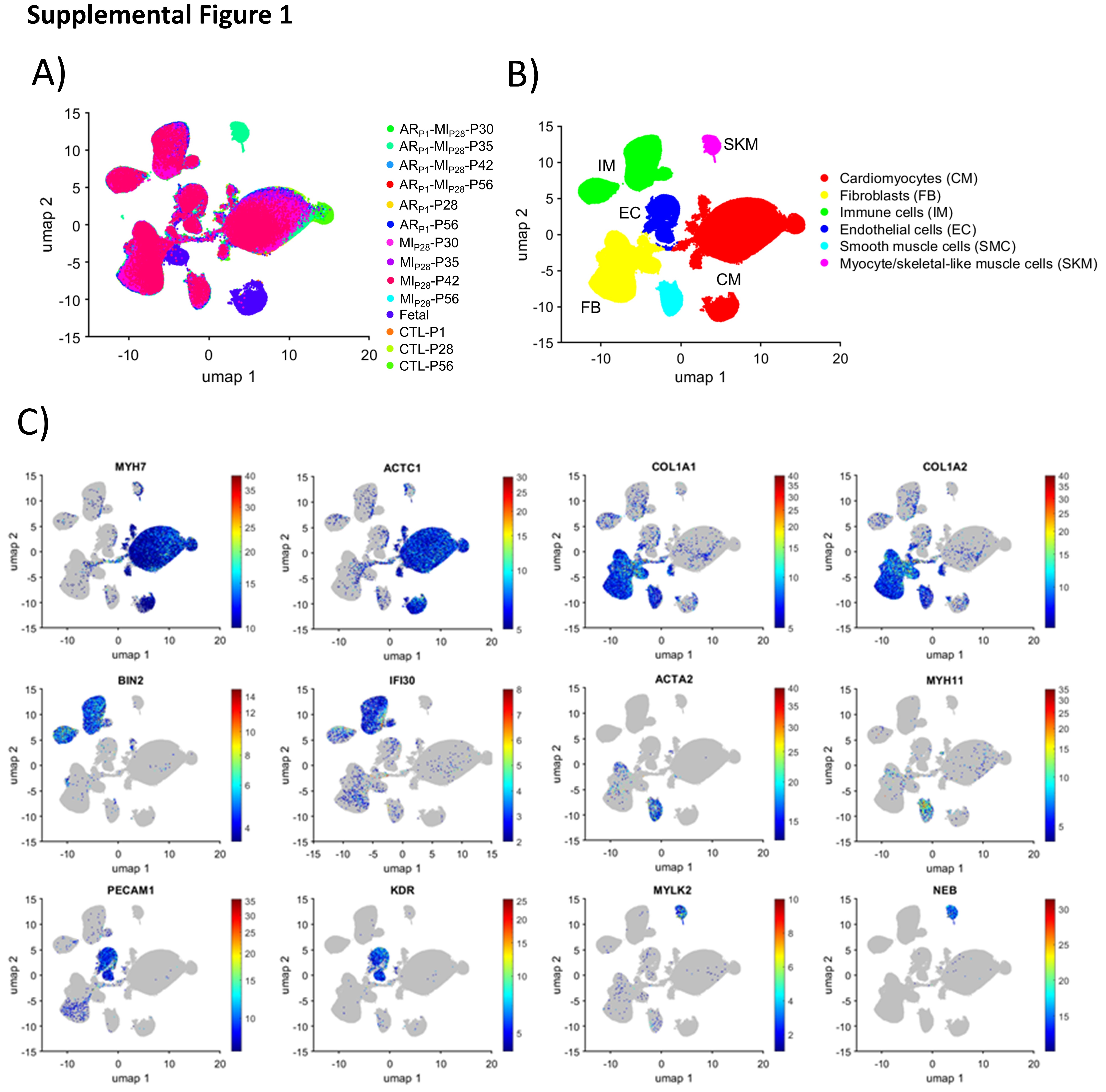

Supplement: Supplementary file 2 [file Image1.JPEG]

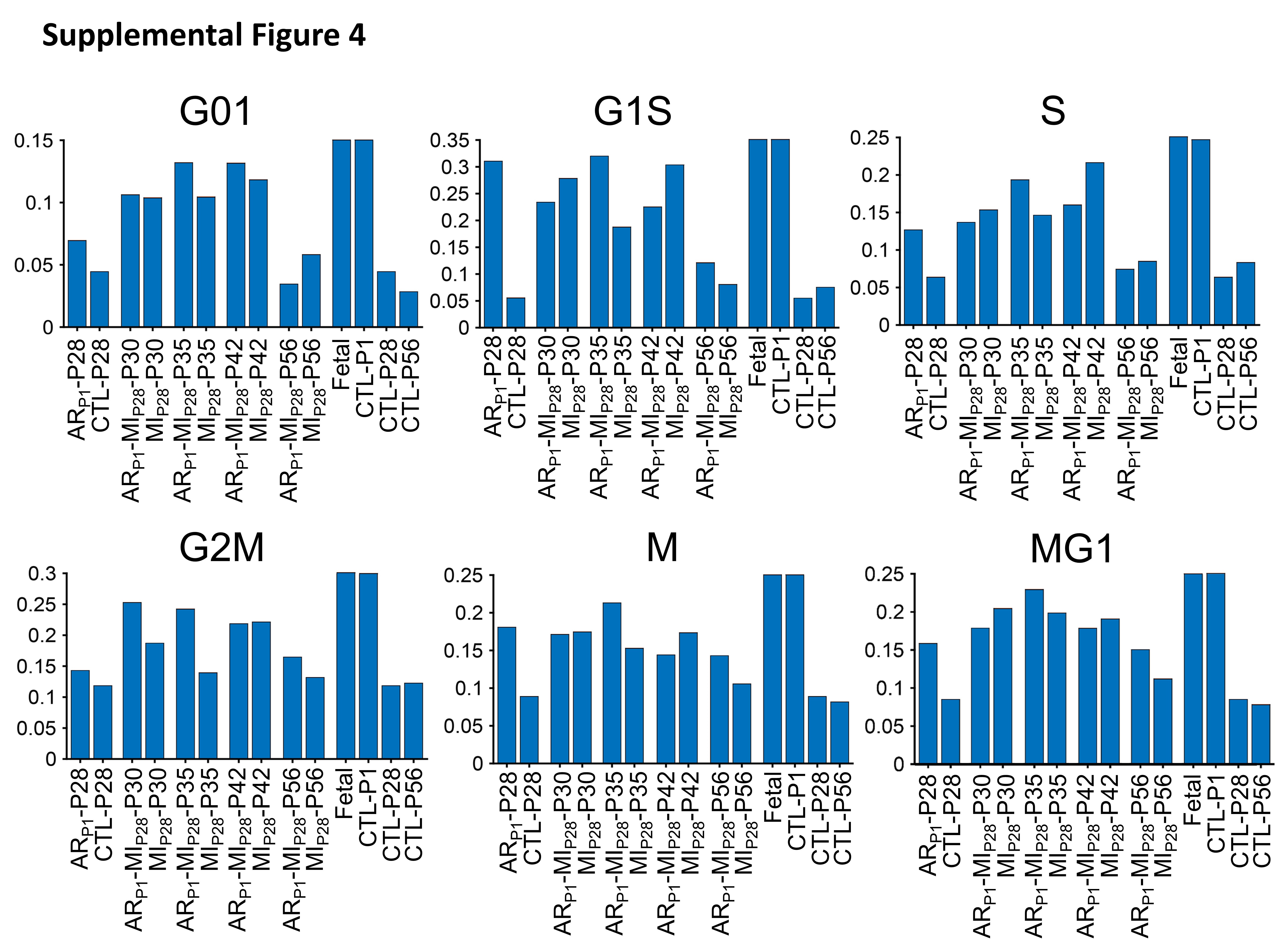

Supplement: Supplementary file 3 [file Image4.JPEG]

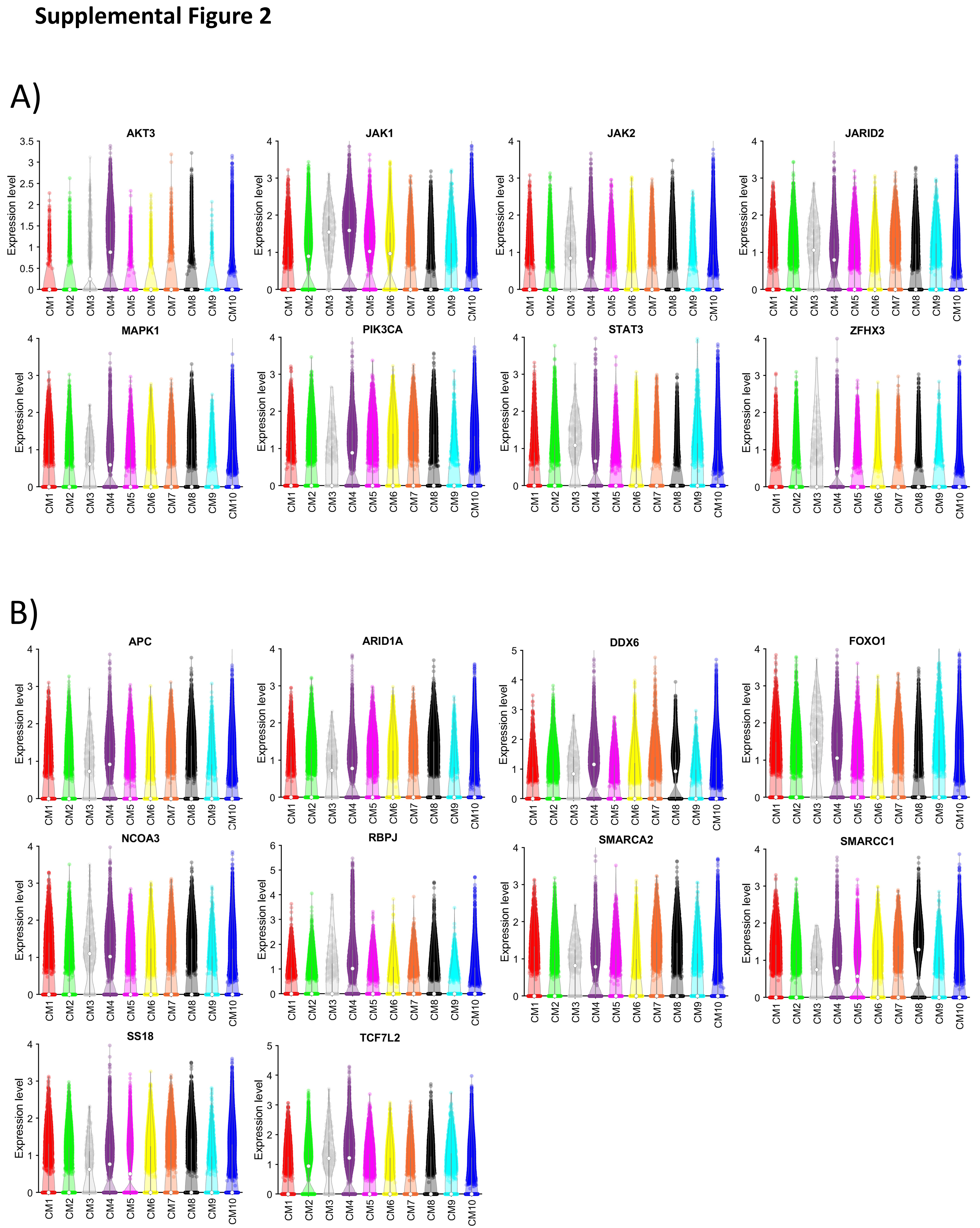

Supplement: Supplementary file 5 [file Image2.JPEG]

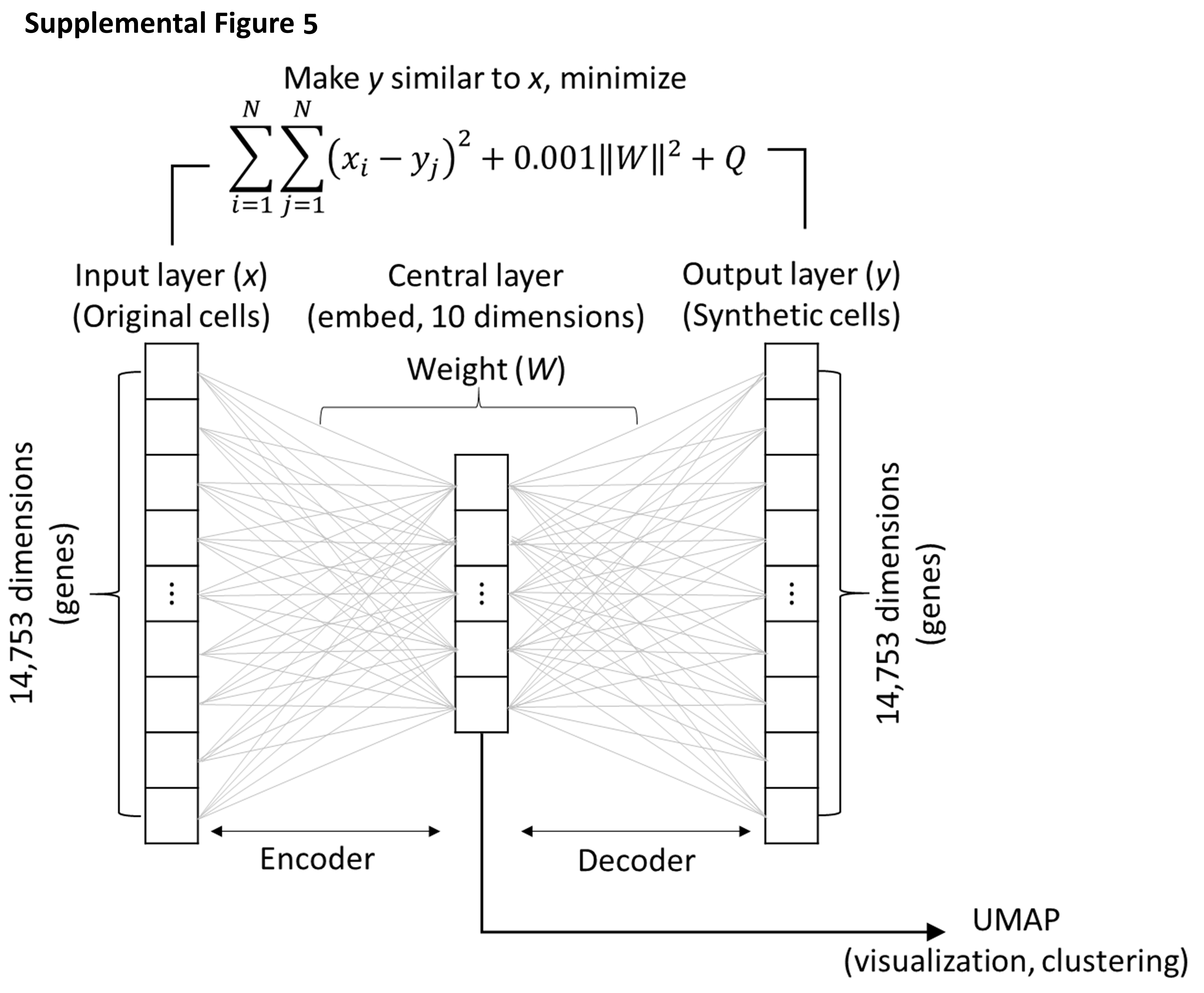

Supplement: Supplementary file 6 [file Image5.JPEG]

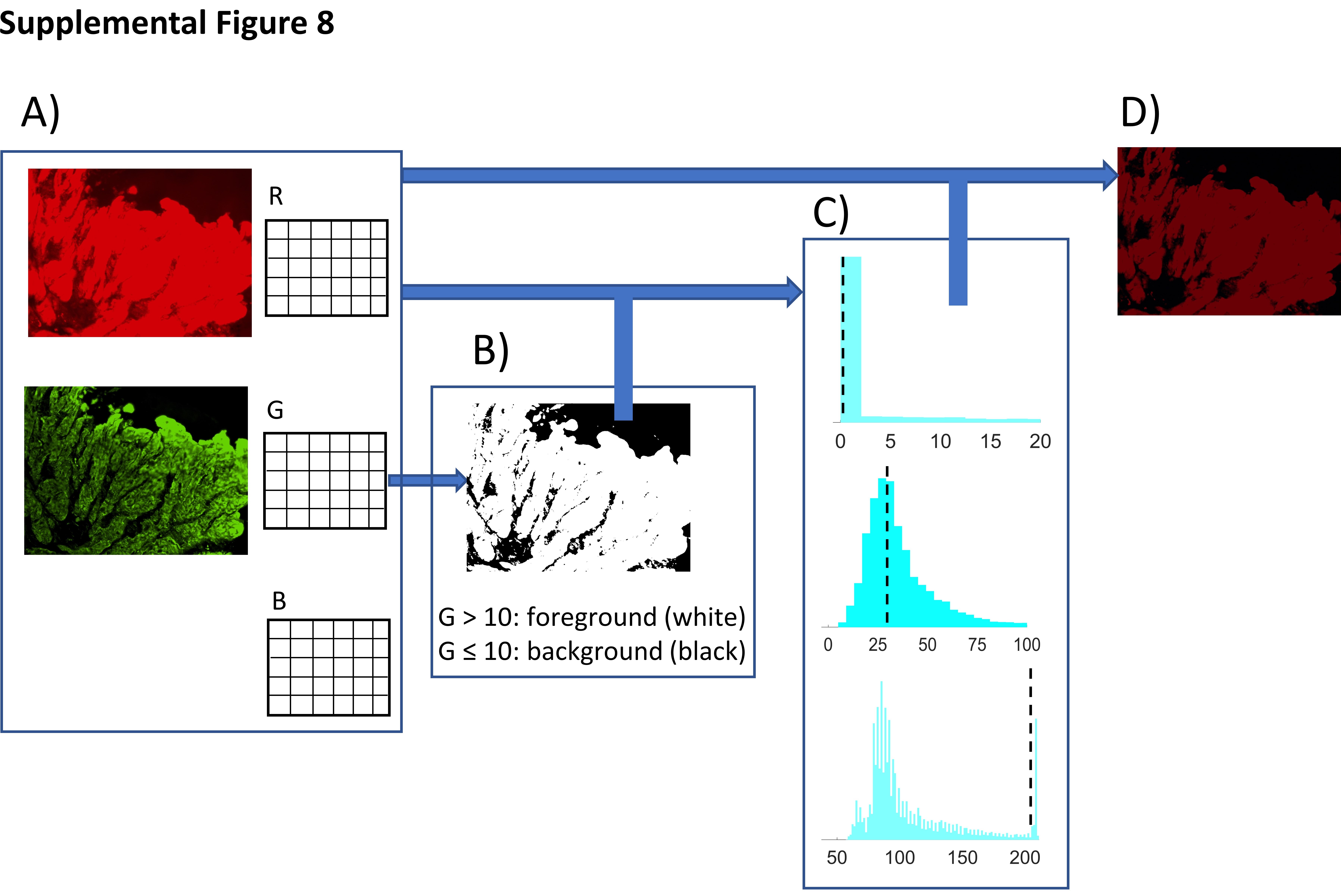

Supplement: Supplementary file 7 [file Image8.JPEG]

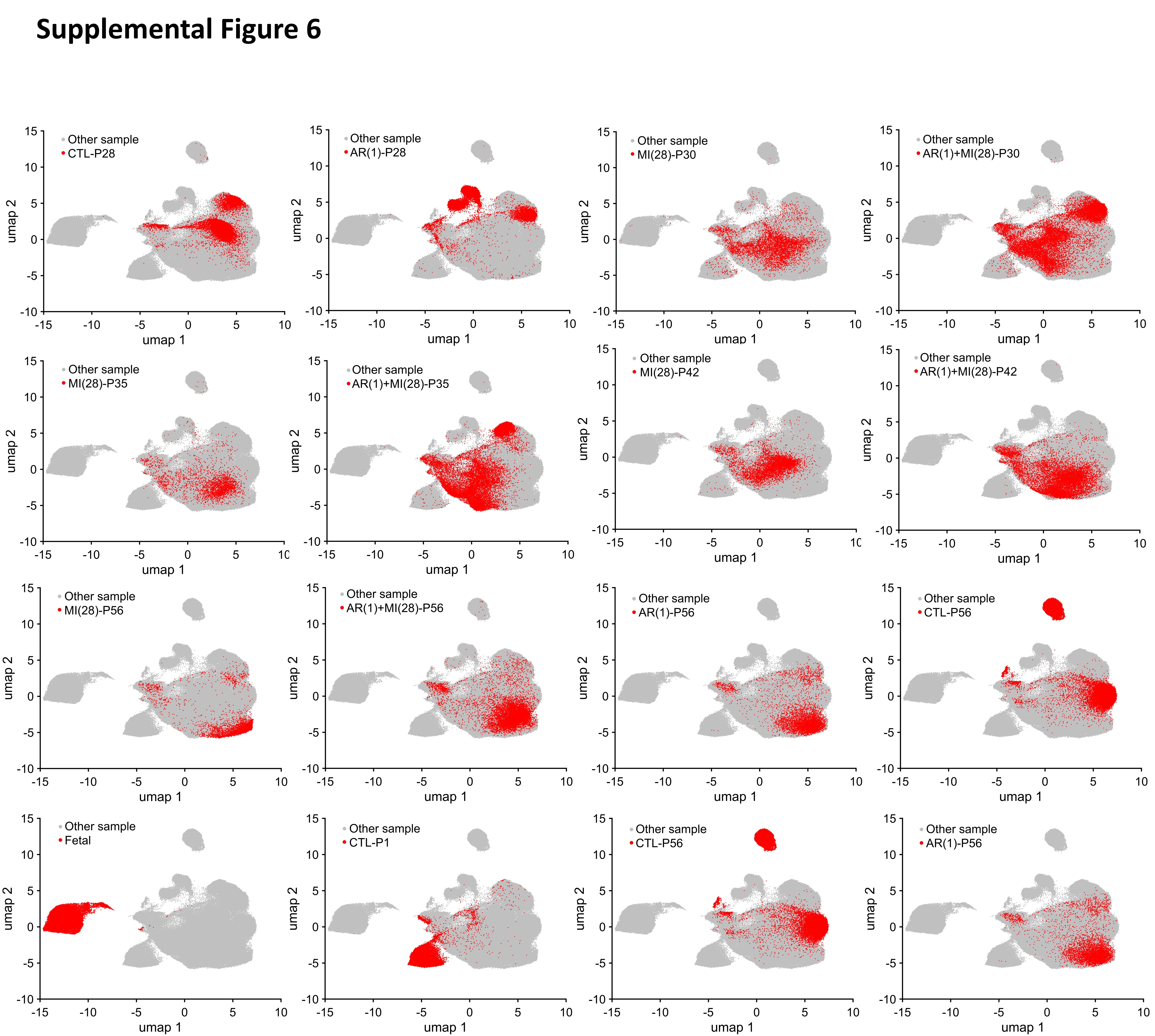

Supplement: Supplementary file 9 [file Image6.JPEG]
