# Supplementary material for: Cardiomyocyte Cell-Cycle Regulation in Neonatal Large Mammals: Single Nucleus RNA-Sequencing Data Analysis via an Artificial-Intelligence–Based Pipeline
Source: Front Bioeng Biotechnol. 2022 Jul 4;10:914450. doi: 10.3389/fbioe.2022.914450 (PMC9289371; doi:10.3389/fbioe.2022.914450)
Supplement: Supplementary file 8 [file DataSheet1.docx]

# SUPPLEMENTAL NOTE 1. Extracting snRNA data for cardiomyocyte

After embedding the normalized snRNA data for all cells with autoencoder, we used Matlab implementation of Umap (https://www.mathworks.com/matlabcentral/fileexchange/71902-uniform-manifold-approximation-and-projection-umap) to visualize and cluster all cells

[reduction, umap, clusterIdentifiers] = run_umap(Z);

Here, Z (283,421 cell/row x 10 column) was the Matlab matrix storing the cell embedding. It results:

- reduction : the 2D visualization of all cells

- clusterIdentifiers: the clustering results using the density-based clustering algorithm (<https://www.mathworks.com/help/stats/dbscan.html>). The result can be reproduced by running

clusterIdentifiers = dbscan(reduction, 30, 0.3);

The cluster result is as follow


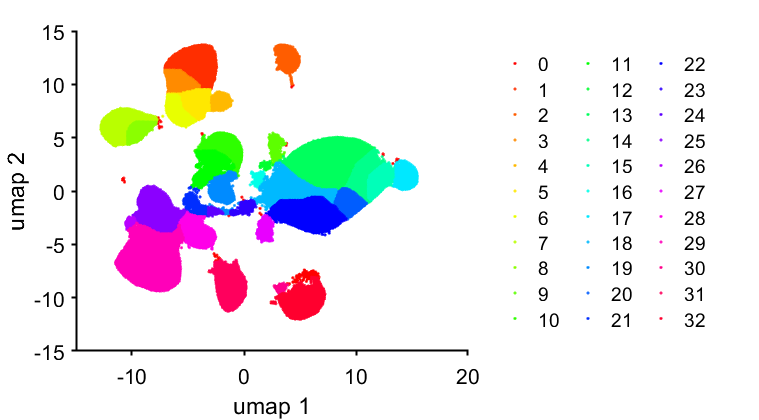


Then, we combined this clustering result and the expression of cardiomyocyte-explicit markers (MYH7 and ACTC1 in Supplemental Figure 1) to determine which clusterIdentifiers were cardiomyocyte. cells having clusterIdentifiers = 17, 15, 14, 13, 20, 18, 22, 32, 30, 9, 12, 16, 27, and 23 were determined as cardiomyocytes.

# SUPPLEMENTAL NOTE 2. Determining cardiomyocyte subpopulations CM1-CM10

After extracting the normalized snRNA for cardiomyocytes (Supplemental Note 1), we built another autoencoder to embed cardiomyocytes. Then, we ran Matlab Umap (https://www.mathworks.com/matlabcentral/fileexchange/71902-uniform-manifold-approximation-and-projection-umap) to visualize and cluster all cardiomyocyte

[reduction, umap, clusterIdentifiers] = run_umap(Z);

Here, Z (129,991 cell/row x 10 column) was the Matlab matrix storing the cell embedding. It results:

- reduction : the 2D visualization of all cardiomyocytes

- clusterIdentifiers: the clustering results using the density-based clustering algorithm (<https://www.mathworks.com/help/stats/dbscan.html>). The result can be reproduced by running

clusterIdentifiers = dbscan(reduction, 30, 0.3);

The cluster result is as follow


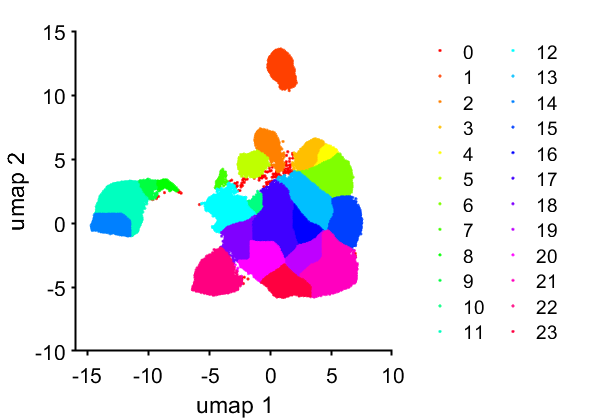


Then, we visualize the distribution of cell in each heart group upon this umap plot as follow


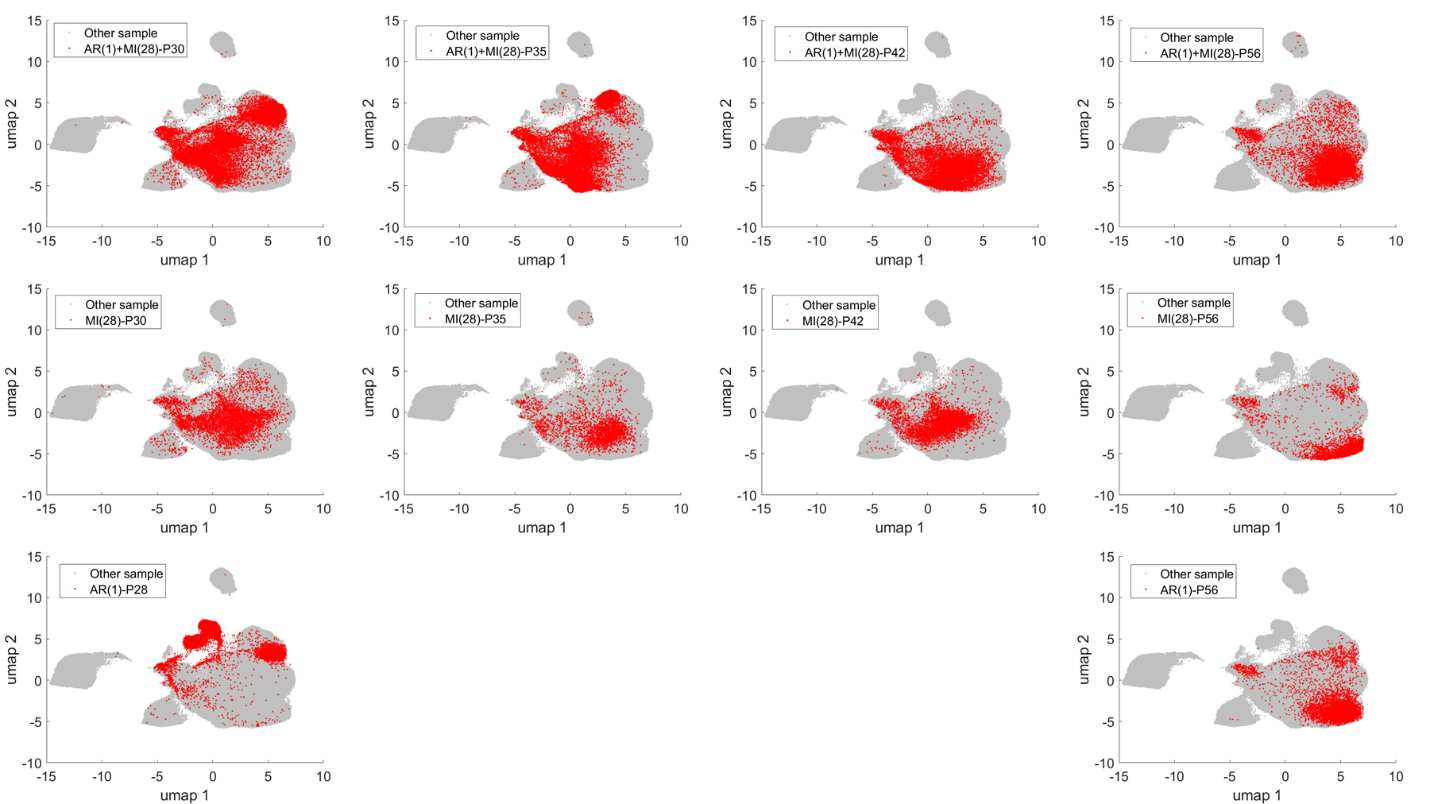

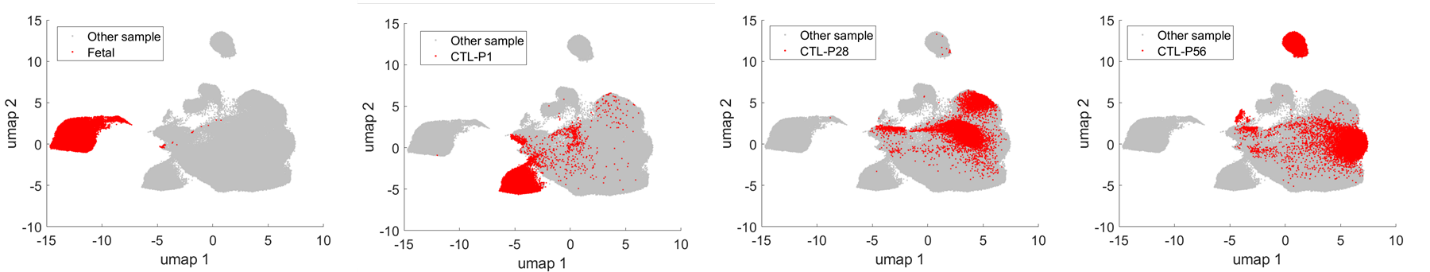


We clearly observed regions that enriched specific groups, especially about the ARp1-MIp28. Therefore, we adjust clusterIdentifiers to identify 10 CM subpopulations:

- CM1 covered cells with clusterIdentifiers = 2 and 5 (ARp1-P28 exclusive)

- CM2 covered cells with clusterIdentifiers = 3, 4 and 6 (explicit for ARp1-P28, ARp1-MIp28-P30, and ARp1-MIp28-P35

- CM3 covered cells with clusterIdentifiers = 7 (very small cluster explicit for CTL-P56

- CM4 covered cells with clusterIdentifiers = 10 and 12 (appear in all groups)

- CM5 covered cells with clusterIdentifiers = 15 (exclusive for CTL-P56)

- CM6 covered cells with clusterIdentifiers = 21 (explicit for injured heart on postnatal day P56)

- CM7 covered cells with clusterIdentifiers = 22 (explicit for CTL-P1

- CM8 covered cells with clusterIdentifiers = 8, 9, 11, and 14 (exclusive for fetal)

- CM9 covered cells with clusterIdentifiers = 1 (exclusive for CTL-P56)

- CM10 covered cells with clusterIdentifiers = 13, 16, 17, 18, 19, 20, and 23 (major cluster among injured heart)
